# Classification prediction of load losses in power stations using machine learning multilayer stack ensemble

**DOI:** 10.3389/frai.2025.1592492

**Published:** 2025-08-07

**Authors:** Bathandekile M. Boshoma, Oluwole S. Akinola, Peter Olukanmi

**Affiliations:** ^1^Johannesburg Business School, University of Johannesburg, Johannesburg, South Africa; ^2^Department of Electrical Engineering Technology, University of Johannesburg, Johannesburg, South Africa

**Keywords:** artificial intelligence, classification, digital transformation, machine learning, multilayer stack ensemble, load loss, power station, explainable Artificial Intelligence (XAI)

## Abstract

Load losses negatively impact the reliability of power stations, leading to plant failures. To support the decision-making of improving plant reliability, we experimented with six machine learning classifiers to find the model combination that produces the best prediction performance, called the Explainable Multilayer Stack Ensemble. We applied a five-year dataset from six power stations. Since the dataset is highly imbalanced with the positive class dominant, class weights are calculated and assigned to reduce bias toward the majority class. The best parameters are determined through a randomized search with cross-validation and applied to train the models. The Explainable Multilayer Stack Ensemble performed better than the individual models, with a further improvement by excluding the Gaussian Naïve Bayes in the second layer since it produced high false negatives. We demonstrate that when handling a highly imbalanced dataset, balanced accuracy, Receiver Operating Characteristics, and Precision-Recall Area Under the Curve provide a more reliable evaluation of model performance than focusing solely on standard evaluation metrics, such as accuracy, precision, and recall. Moreover, by excluding a poor-performing classifier from ensemble, we optimized the prediction process, and further enhanced overall performance.

## 1 Introduction

Digital transformation is changing the traditional ways in which organizations manage their operations. In particular, the electricity industry is experiencing a substantial shift influenced by advancements in digital technologies. For instance, the rapid growth of artificial intelligence (AI) has led to using digital technologies such as machine learning to optimize operations and maintenance (Ugboke et al., 2024). Through the combination of predictive analytics and advanced algorithms, utilities can make informed decisions to improve the reliability of their plants (Aghahadi et al., 2024) by taking advantage of the well of data they have at their disposal. While big data has become essential for decision-making (Čačković and Popović, 2016), some organizations still struggle to harness its potential and gain a competitive edge. According to Shuvo and Yilmaz (2020), incorporating AI into operations improves system efficiency and reliability. One of the issues facing the electricity industry is how to enhance the stability of generating plants (Wenyu et al., 2021). Electricity companies such as Eskom are grappling with load losses, which reduce the electricity output a power station can generate. To eradicate load losses, a robust maintenance regime is required. In many power companies, the maintenance procedures typically involve regular routines based on specific operational timeframes (Ukkelberg, 2018) recommended by the original equipment manufacturer (OEM). However, given the tight nature of the grid with more demand for electricity than available supply, in the recent past, it has become a challenge for power utilities like Eskom to perform planned outages, resulting in a backlog of maintenance in most power stations. This constraint has had an adverse impact on the reliability of the power stations, evident from the increase in unplanned capability loss factor (UCLF).^1^ There is growing research on the electricity industry. Nevertheless, a significant portion of this research primarily focuses on power distribution, power transmission, and renewable energy sources. As a result, there exists a noticeable gap in research addressing load losses in power stations, particularly in relation to the application of machine learning techniques. Given the challenge of declining EAF faced by power stations due to the increase in load losses caused by UCLF, traditional management of load losses, such as manual spreadsheets, falls short in identifying hidden patterns in the dataset. Furthermore, since machine learning is a black box, most machine learning models do not provide transparency or interpretability on how they arrive at their decisions. In this paper, we present a novel model called Explainable Multilayer Stack Ensemble (EMSE), which utilizes machine learning ensemble models and Explainable Artificial Intelligence (XAI) using Shapley Additive Explanations (SHAP) to identify load losses in power stations. The primary goal of this study is to enhance the comprehension and interpretation of the classification process while identifying the critical elements that influence load loss prediction, to help power utilities reduce load losses, especially those that result in UCLF. By applying power station plant data from historical load loss events to a machine learning multilayer stack ensemble, our approach aims to empower power utility managers with actionable information, ultimately enhancing plant reliability and facilitating empirically informed decision-making interventions.

The intelligent prediction of load losses will ensure that power stations become reliable by proactively focusing on plants that urgently require outages, thereby scheduling a planned outage in advance that leads to Planned Capability Loss Factor (PCLF) instead of UCLF. Therefore, in this study, load losses are classified as UCLF or PCLF. The priority focus of this study is on the load losses contributing to UCLF, which is our label of interest. By predicting load losses in advance, our contribution is to help the management of power utilities perform a quick evaluation of the plants needing attention before failure occurs and prioritize them for the planned outage schedule. This proactive approach, which is a significant step forward in power plant management, can significantly enhance decision-making, enabling power utilities to reduce the UCLF and improve overall plant reliability. Hence, the objective of this study is to explore several machine learning models using a multilayer stack ensemble approach to find the best ensemble with improved classification performance to aid in the decision-making of prioritizing planned maintenance for the stations at risk of failing.

The main contributions of this paper are:

- Development of a robust load loss prediction model (EMSE) for classification of load losses.
- Integration of XAI using SHAP approaches for model interpretability and understanding of the underlying decisions for the model in predicting load losses.
- Empirical validation through a secondary five-year dataset of historical load loss events from six power stations, demonstrating the model’s feasibility.
- Superior performance of the EMSE compared to baseline models, achieving a prediction accuracy of 96%, a precision rate of 0.996801, and a balanced accuracy of 0.881929.
- Improved early identification of UCLF load losses, supporting power station managers and plant personnel with actionable information that aids in decision-making for prioritizing plants that need planned outages.

The paper is structured in seven sections. In Section 2, we provide the theoretical foundation of this study, explaining the relevant key concepts. Section 3 briefly delves into related work to highlight the novelty of this research. Section 4 presents the approach used for the classification tasks employing machine learning techniques. In Section 5, we compare the results obtained from the different experiments, and in Section 6 discuss the results. Finally, Section 7 encapsulates the conclusion and puts forth recommendations for future research.

## 2 Theoretical basis

In this section, we briefly explore the fundamental concepts that form the backbone of this research. We start by explaining the key terms related to the concepts used to provide readers with an enhanced understanding of the basis of our research.

### 2.1 Digital transformation

Digital transformation is a catalyst for change across various spheres, particularly within the business context, and it impacts all facets of human existence through the utilization of technologies (Kraus et al., 2021). Digital transformation is leading to substantial changes in businesses (Schwertner, 2017) as organizations are adopting technologies to improve the efficiency of their business operations (Hustoft and Weber, 2019) and take advantage of the value creation benefits (Morakanyane et al., 2017). Consequently, integrating digital technologies as part of the transformation can introduce new processes or alter existing ones (Hagberg et al., 2016). This study examines the potential of adopting digital transformation through the application of machine learning to enhance the reliability of power stations, particularly in facilitating strategic decision-making regarding the scheduling of planned outages.

### 2.2 Load losses

A load loss refers to the inability of a power plant to generate electricity at its designated maximum continuous rating (MCR) (Powertech, 2008) for several reasons, including but not limited to equipment faults (Marx et al., 2021), technical malfunctions of power plant components (Gils et al., 2018), and plant failures due to conditions that arise as a result of poor plant maintenance or defects. Irrespective of their causes, load losses fall into the broad categories of either planned or unplanned and they are categorized as partial or full load losses (Marx et al., 2021). Planned load losses are incurred while the plant is down for planned maintenance. Unplanned load losses, as the name implies, are unplanned. Partial load losses are energy losses that result in the power station being unable to operate at full capacity. Conversely, full load losses are major plant incidents that result in a full plant breakdown or reduction of a significant amount of load. Examples of full load losses include unit trips, outage slips, and boiler tube failures. The amount of time incurred and the amount of electricity that cannot be produced as a result of a load loss are known as hours loss and Megawatt loss (MW Loss), respectively. The plant downtime to address the load loss or fix the cause of the load loss is called an outage, and it may be planned or unplanned (Ogieva et al., 2015). For a power utility like Eskom, an outage is considered a planned outage if it is scheduled at least 4 weeks in advance1. If the outage occurs reactively or is scheduled in less than 4 weeks before it happens, it is classified as an unplanned outage. Thus, plant failures and other equipment malfunctions are undesirable as they result in load losses that reduce the amount of electricity in a power system, requiring repairs or fixing that result in outages. Therefore, this study explores the various machine learning models that can enhance the proactive management of load loss by enabling advance predictions, thus allowing power stations to take timely measures for planned outages.

### 2.3 Big data

A vast quantity of data is being produced by many organizations. This substantial amount of data, also known as ‘big data’, lacks significance until relevant information is extracted from it (Neeraj and Maurya, 2020). Big data can be defined as the collection of large volumes of data from diverse sources, which is then analyzed to generate fresh insights and gain competitive business advantages (Čačković and Popović, 2016). Due to its large scale, complexity, and multidimensionality, big data cannot be effectively analyzed using basic computational techniques. As a result, machine learning methods for data mining are necessary (Neeraj and Maurya, 2020). Data mining involves uncovering hidden information such as patterns, interactions, correlations, groups, features, outliers, and more from the data (Neeraj and Maurya, 2020; Jackson, 2002). This study is unique in that it utilizes a real-life dataset derived from historical load loss incidents of power stations recorded over the past 5 years, thereby providing a robust foundation for analysis and enhancing the validity of its findings in the context of load loss prediction and management in power generation.

### 2.4 Machine learning

One commonly utilized approach in data analysis is machine learning (Tareq and Davud, 2024). Machine learning is a field of data science and a subset of AI that focuses on the development of algorithms (Nist, 2015) to enable computers to learn from data in order to improve (Mitchell and Mitchell, 1997). Machine learning encompasses a variety of methods that can recognize data patterns and leverage these patterns to predict future results (Jolly, 2018). Through the learning process, machine learning gains experience without requiring specific programming for each new task (Brossa Dachs, 2018; Samuel, 2000). The three types of learning used by machine learning include supervised, unsupervised, and reinforcement learning (Jolly, 2018). In supervised learning, the data comes with a set of labels or a numerical target variable, typically associated with a single feature or attribute, commonly referred to as the target variable (Jolly, 2018). In supervised learning, the machine learning model is trained on a labeled dataset called training data for a prediction task and then assessed on an unfamiliar dataset known as a testing set or validation dataset (Nti et al., 2021).

In contrast to supervised learning, unsupervised learning does not use predetermined labels, instead it employs algorithms to identify patterns in data without an outcome or target variable, allowing for the clustering of unlabeled data into discernible groups to which labels can be subsequently applied (Jolly, 2018). The primary distinction lies in the fact that supervised learning is used to train models for predicting future events, whereas unsupervised learning is utilized for pattern discovery (Brossa Dachs, 2018). Hence, supervised learning requires labeled datasets for training, whereas unsupervised learning does not need labeled data (Sharifzadeh et al., 2021).

Conversely, reinforcement learning involves the agent engaging with the environment to acquire knowledge and formulate decisions, with the environment providing rewards or transitioning to a new state in response to the agent’s actions (Chakole et al., 2021; Li, 2017; Li et al., 2022; Meng and Khushi, 2019; Pendharkar and Cusatis, 2018; Sahu et al., 2023; Xu et al., 2022; Zhang et al., 2022).

Since machine learning algorithms enable computers to make intelligent predictions, which can be used for decision-making (Management and Brown, 2021; Nazari-Heris et al., 2021; Vaish et al., 2021), data is the critical component used as a basis for learning and making predictions (Management and Brown, 2021; Marsland, 2011; Sharifzadeh et al., 2021).

Machine learning serves three functions: descriptive to explain what happened, predictive to predict what will happen, and prescriptive to make recommendations about actions to take (Management and Brown, 2021). Since the primary objective of a machine learning model is to ensure that its predicted outcomes correspond closely with actual results; thus, the evaluation of the model’s performance is crucial (Plevris et al., 2022). Consequently, the integration of machine learning into current operational frameworks has the potential to enhance efficiency. However, this integration requires the availability of high-quality data (Ukkelberg, 2018).

## 3 Literature review

In this section, we consider existing literature that used machine learning to predict load losses in the electricity industry. In the discussion of the existing literature, we highlight the differences in the machine learning tasks, domains of application, and the specific focus of each study in order to demonstrate the distinctive contributions and approaches of our own research.

Studies on predicting load loss in the electricity industry are becoming more common (Abaas et al., 2022; Bush et al., 2021; Chadaga, 2023; Dalal et al., 2021; Grando et al., 2019; Han-Min and Chae-Joo, 2022; Hou et al., 2023; Li et al., 2021; Mahgoub, 2021; Mahgoub et al., 2020; Majzoobi et al., 2017; Nakas et al., 2023; O’donnell and Su, 2023; Omogoye et al., 2023; Shuvro et al., 2019; Uwamahoro and Eftekharnejad, 2023; Wang et al., 2023). Many of these studies focus on power distribution (Dalal et al., 2021; Grando et al., 2019; Han-Min and Chae-Joo, 2022; O’donnell and Su, 2023; Omogoye et al., 2023), while others concentrate on power transmission (Bush et al., 2021; Omogoye et al., 2023; Shuvro et al., 2019) and some on renewable power sources (Hou et al., 2023; Nakas et al., 2023; Wang et al., 2023). Most of these studies are concerned with prediction of cascading failures (Chadaga, 2023; Li et al., 2021; Mahgoub, 2021; Mahgoub et al., 2020; Nakas et al., 2023; Shuvro et al., 2019; Uwamahoro and Eftekharnejad, 2023).

The research by Majzoobi et al. (2017) employed an Adaptive Network-Based Fuzzy Inference System (ANFIS) to forecast the lifespan of transformers, which is part of the realm of predictive maintenance for electrical infrastructure. ANFIS is a blend of Artificial Neural Networks (ANN) and fuzzy inference systems, and the researchers used numerical simulations in MATLAB to predict the hourly remaining life of transformers. This task falls within the scope of regression in machine learning since it entails forecasting a continuous value, specifically the remaining lifespan of a transformer. Conversely, our research focuses on predicting losses in power stations, encompassing multiple pieces of equipment, as opposed to being limited to the prediction of losses for only one equipment, namely a transformer. Additionally, while Majzoobi et al. (2017) implemented their model in MATLAB, we develop and train our models using Python.

A study by Grando et al. (2019) presents a classification of short-circuit faults in electrical distribution networks using machine learning. They utilized data from Phasor Measurement Units (PMUs) installed along the network to simulate instances of faults. They applied Linear Discriminant Analysis (LDA), k-nearest Neighbors (KNN), Support Vector Machines (SVM), Artificial Neural Networks (ANN), and Decision Trees (DTs). The uniqueness of our study compared to that of Grando et al. (2019) is that while they conducted a study on classifying faults, we not only classify faults but all load loss types. Additionally, our classification task is for power stations, whereas theirs is in a distribution network.

A similar study by Shuvro et al. (2019) employed various machine learning algorithms to predict and classify cascading failures into three categories, namely, no cascade, small cascade, and large cascade. Additionally, they used linear regression to predict the number of failed transmission lines and the amount of load shedding during a cascade. In their study Shuvro et al. (2019) utilized operating parameters such as loading levels, capacity of failed lines, and topological parameters as features for the machine learning models. While their study applies to a power transmission grid, we use different features from a power station environment, and our classification task is based on load losses of UCLF, Other Capability Loss Factor (OCLF), which contribute toward the unplanned outages, and Planned Capability Loss Factor (PCLF), which contributes toward planned outages.

A paper by Bush et al. (2021) created a novel topology-based system for rapid synthetic regulation and contingency reserve services for the power grid. Their machine learning task involved classifying and predicting impact metrics in power systems using a neural network architecture and local topological measures to evaluate the impact of different power contingencies and predict unserved load. However, they used only one machine learning model, namely the neural network, while in our study, we experimented and compared multiple classifier models. An additional uniqueness of our study in relation to theirs is that their prediction application is in a transmission network, while ours is in power stations.

A comprehensive study by Dalal et al. (2021) conducted a day-ahead forecasting of grid loss in a distribution network using a machine learning system with 24 different models to generate forecasts for three sub-grids. The focus of their task was regression, and the domain of application is the energy distribution network. In contrast, our study involves a dataset encompassing 5 years of data, whereas Dalal et al. (2021) worked with a smaller dataset containing less than 2 years of data. Additionally, the application of Dalal et al. (2021) was set in a distribution network, whereas our application is in a generation domain. Furthermore, their prediction task revolved around regression, while ours is a classification problem.

Another study conducted by Hou et al. (2023) focused on developing a multi-stage hybrid energy management strategy utilizing machine learning algorithms for a regression task to predict energy abandonment and load loss during the scheduling of renewable energy. A study by Hou et al. (2023) employed various machine learning models, including Extreme Learning Machine, BP Neural Network, Linear Regression, Support Vector Regression, K-Nearest Neighbor, Random Forest, AdaBoost, Gradient Boosting Decision Tree, Bootstrap Aggregating, and Extra Tree. The difference between their study and ours is that their prediction application is for energy management for microgrids in a peak-load energy landscape, while ours is in a base-load energy domain.

An interesting study by Wang et al. (2023) deals with an online load-loss risk assessment method for power systems using stacking ensemble learning. They first used a traditional load-loss risk assessment method based on power flow analysis to create risk samples. Then, they trained the stacking ensemble on four different machine learning models, namely Support Vector Regression (SVR), Extremely Randomized Trees (ET), Extreme Gradient Boosting (XGBoost), and Elastic Network. Additionally, they utilized Particle Swarm Optimization algorithms to optimize the model parameters. The machine learning task involves regression in a stochastic environment involving wind energy, while our prediction involves classification in a base-load application.

Research by Yeardley et al. (2022) has employed machine learning for the prediction of maintenance task scheduling. However, a significant limitation of their approach lies in the reliance on synthetic data generated through computer simulations, rather than utilizing actual operational data. Although the authors contend that access to empirical data is constrained, the primary concern remains that synthetic datasets may not accurately reflect the conditions present in real production environments. This gap warrants to be addressed, as the dynamics observed in a simulated context may differ markedly from those in real world scenarios, thereby limiting the generalizability of the findings.

The studies mentioned above have established a solid groundwork using AI more especially machine learning. However, these existing studies highlight the need for more research on predicting load losses within power generation in comparison to the extensive research in power distribution, transmission, and peak-load domains. In addition, the need for real world data remains a gap and is critical in ensuring that predictive models accurately reflect actual operational conditions, thereby enhancing their reliability and effectiveness in informing maintenance strategies and decision-making processes. These gaps underscore the need for further research and development, specifically in the classification of load losses in power stations. Additionally, our study differs from existing literature in several ways. Firstly, our data source comprises secondary data from power stations. Secondly, our study utilizes real-world load loss events data from multiple plant areas and equipment. Thirdly, the focus of our study is on power generation.

In the next section, we will discuss the materials and methods employed to achieve the objectives of this study.

## 4 Methods and materials

**Figure 1:**
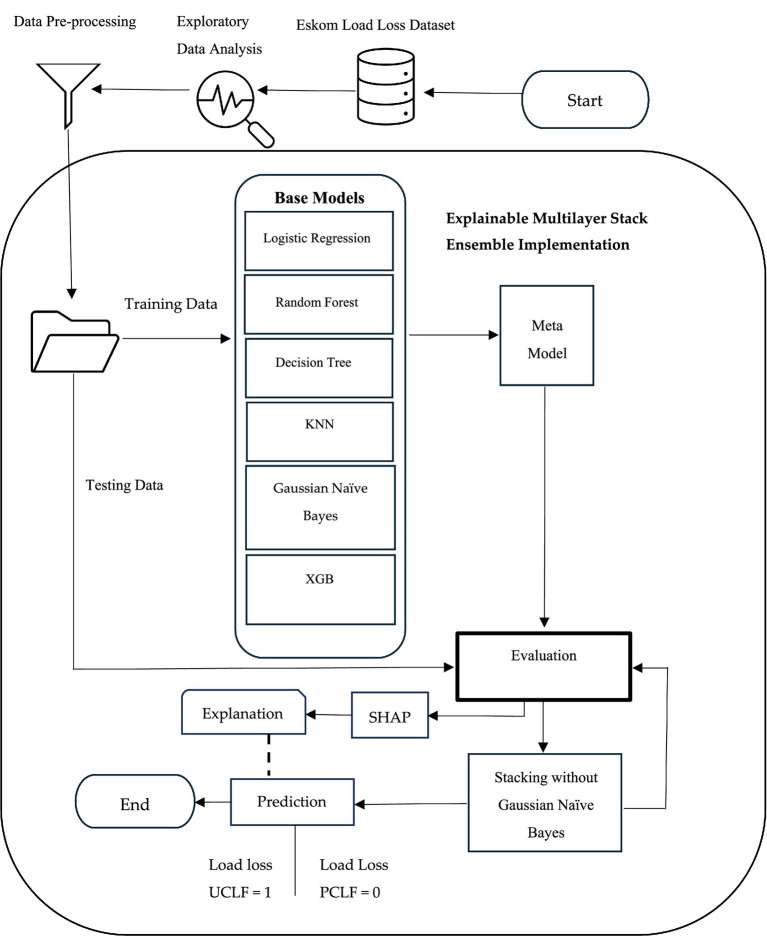
Conceptual framework.

We introduce a new model (EMSE) for predicting load losses in power stations. This section conveys the techniques, tools, and methodologies employed to accomplish the objective of the study. Figure 1 illustrates the main steps of the proposed EMSE model, which depicts the various stages involved in developing the proposed model for load loss classification. It highlights exploratory data analysis, data pre-processing, split for the training and testing data, the base models used in creating the EMSE, the evaluation in achieving accurate and reliable predictions, and interpretation using SHAP.

### 4.1 Dataset

We obtained a real-life dataset from Eskom, the largest power utility in South Africa. The dataset contained secondary data on historical load losses from six power stations: Arnot, Camden, Duvha, Kendal, Lethabo, and Medupi. The load loss records span over 5 years, from the 2018 financial year (FY), which starts in April 2017, to the 2022 financial year, which ends in March 2022, providing a large and rich set of observations. The dataset contained 97,986 rows of data, and 37 columns, with mixed categorical and continuous variables. According to Wu and Hargreaves (2021), mixed data with both numeric and categorical attributes is common in real-world applications, but many algorithms cannot handle this and can only process singular types of data, either numeric or categorical.

### 4.2 Exploratory data analysis

To gain insights into our dataset, we conducted exploratory data analysis (EDA) utilizing a univariate graphical approach to identify the distribution of each category across the entire dataset. The original dataset consists of 64,300 data records comprising four categorical features including Station Name, Unit, Plant Description 1, and a target variable called Classification that contains three variables, namely: UCLF, OCLF, and PCLF. In addition, there are two numeric features known as MWH Loss and MW Loss.

**Figure 2:**
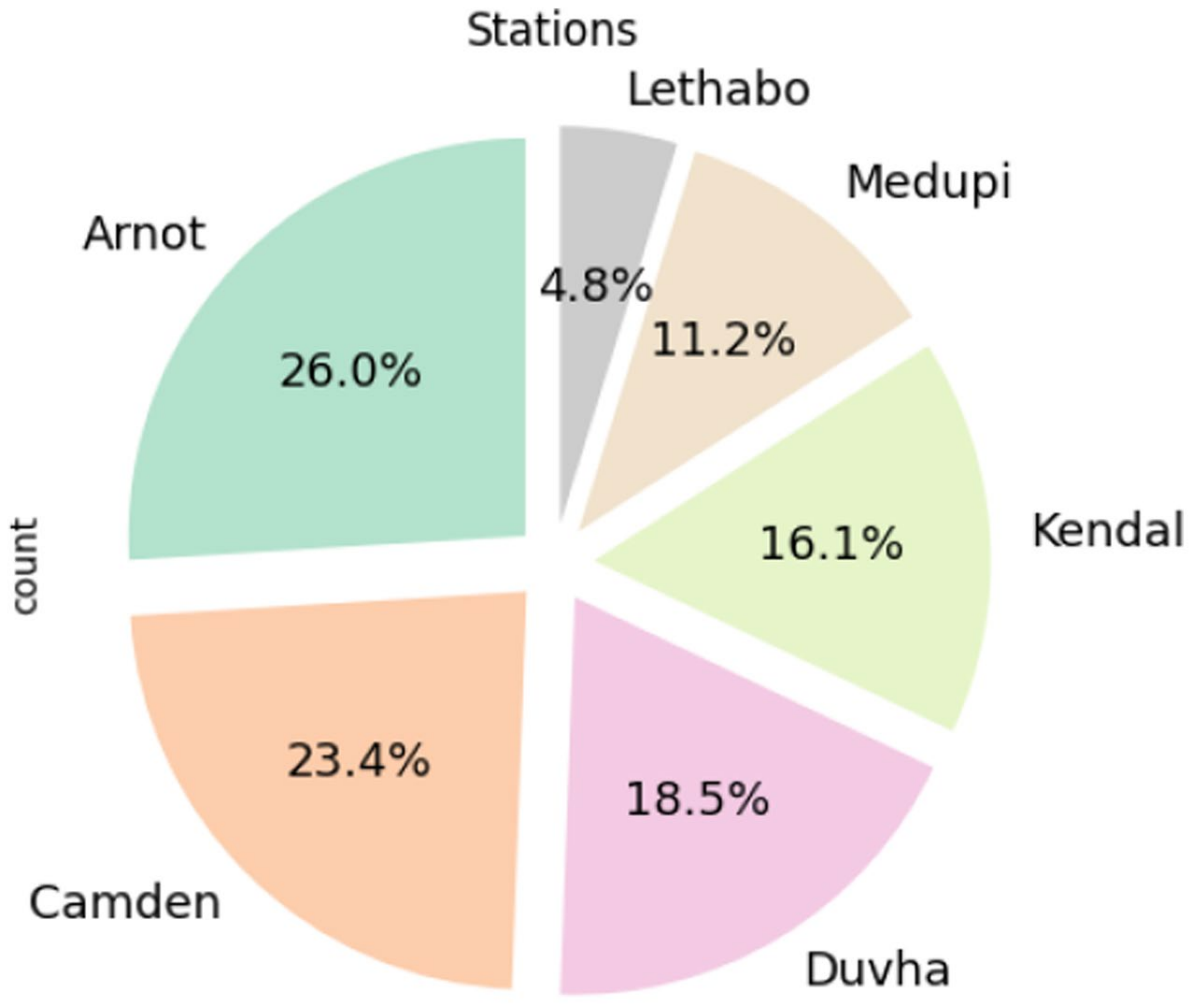
Prevalence of load losses in power stations.

**Figure 3:**
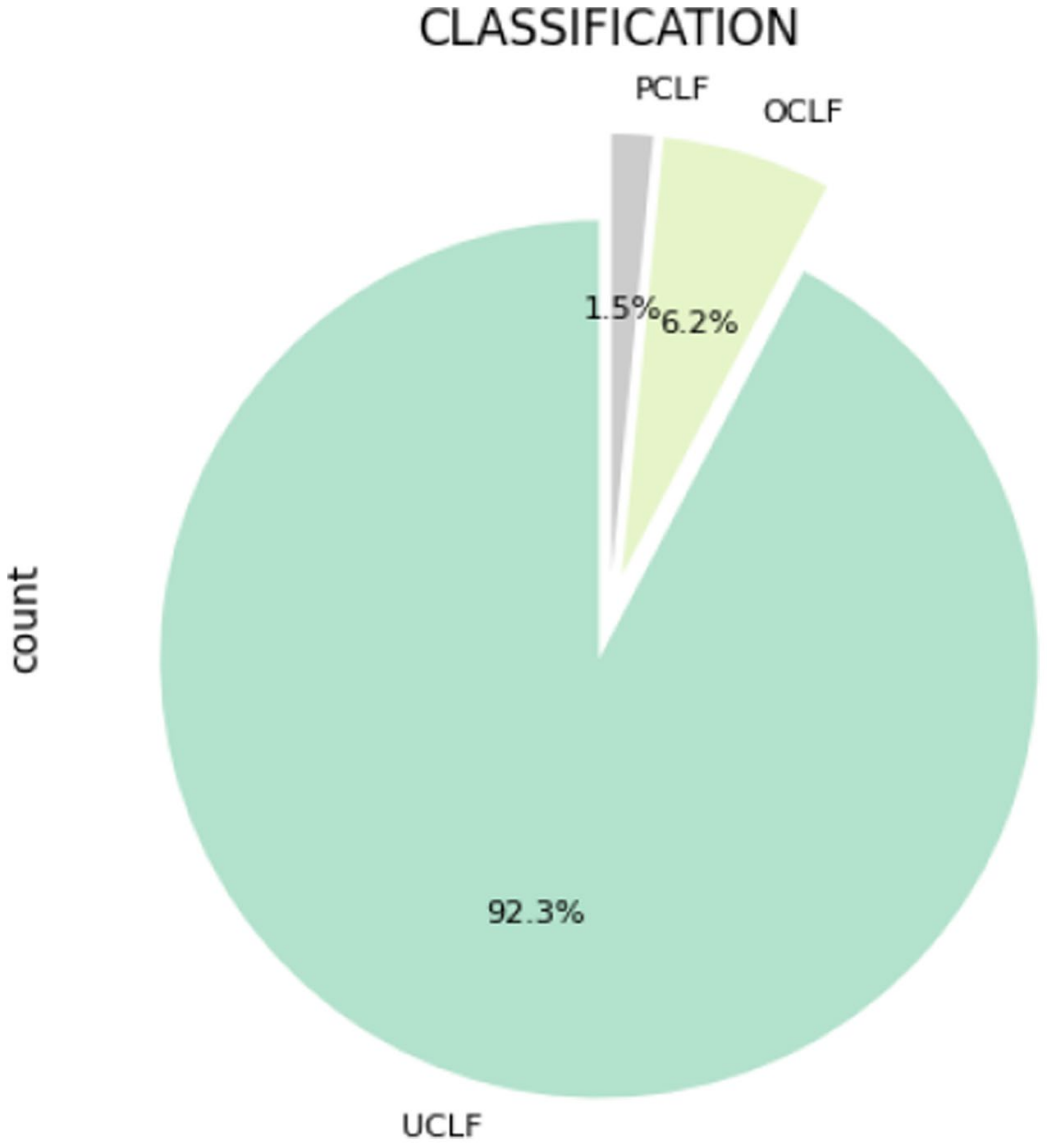
Composition of UCLF, OCLF, and PCLF within load losses.

According to Figure 2, load losses are more prevalent at Arnot, Camden, and Duvha. Figure 3 shows the composition of load losses and displays the distribution of UCLF, OCLF, and PCLF within the load loss events. The composition comprises 92% UCLF, 6.2% OCLF, and 1.5% PCLF, with UCLF being a predominant class.

### 4.3 Data pre-processing

The quality of data is essential for the performance of learning algorithms (Ruiz-Chavez et al., 2018). Therefore, pre-processing the data to prepare it for data mining was a necessary step. Given the imperfect nature of real-world data (García et al., 2016), we undertook a comprehensive data-cleaning process. By running the imported dataset in Pandas in a Python environment, we identified the unique properties in the dataset.

#### 4.3.1 Overlapping and redundant features

The data contained overlapping features and redundancy present in the form of duplicated data in more than one column presented as different labels. An example is the MW hours loss, which is a mathematical outcome of MW loss and Hours loss. We dropped the Hours loss column since it was already catered for in the MW hours loss column. We also dropped the Truncated start date and the Truncated end date, which are similar to the Actual start date, and the Actual end date, respectively.

Removing unnecessary information that did not contribute to solving the problem of this study was a crucial step we undertook. This step was essential in maintaining the focus and purpose of our research. Features such as the Allocation ID, Station Event ID, Shutdown Code, Energy Code, and Outage Schedule ID were removed since they served no purpose for the study. In addition, some information from the target features, such as events like ramping up, cold reserve, spinning reserve, and outage slip events, were also removed. Although these events are recorded as load losses, they do not form part of the scope of this study as they do not occur as a result of plant defects, equipment faults, and plant breakdowns. Therefore, we removed them as they were irrelevant to fulfilling the research objectives for this study.

#### 4.3.2 Ambiguous data

We identified ambiguous data, which occurred as a result of plant operators from different stations recording the same load loss reason differently using free text. Examples of ambiguous data in the load loss file include the inconsistent spelling of the same word, such as primary air, which in some instances is recorded as PA, in others P.A. Another example is feeder, which some plant operators write as feeder, others fdr, and in other cases misspelled as feeeder. Another observed ambiguity was the use of ‘vanes@ max open,’ ‘vanes maximum open,’ and ‘vanes open maximum.’ In addition, we found instances where GM/, G/M, gen motor, and generator motor were recorded interchangeably. Another classic case noted was the term ‘out of commission,’ which appears in the data as o/c, o.c, not available, unavailable, out of service, and o/s. Such ambiguity can result in the loss of meaning, misinterpretation, lack of clarity, and analysis errors. We then decided on a standard naming convention by grouping similar reasons into themes to resolve the ambiguity of the non-standard recording. For example, a theme called ‘Coal conveyor belt damaged, faulty, maintenance’ was allocated to reasons relating to conveyors that were out of commission, failed, being spliced, or repaired. Another example was allocating the theme ‘PA fan vanes maximum open’ to denote any PA fan-related reasons, which were recorded as vanes@ max open,’ ‘vanes maximum open’, ‘vanes open maximum’, or ‘PA fan vanes 100% open’. ‘Bearing temps high’, ‘brg temperature high’, and ‘bearing temp spiking’ were given a theme called ‘bearing temperature high.’

In addition, we identified and corrected spelling errors for words such as condenser, which on several occasions was misspelled as condensor; mill trunnion, which was misspelled as mill trinoin; and flue gas, which was misspelled as flu gas. Errors in the spelling of text data can cause machine learning algorithms to be unable to process the structure of the text and thus lose the correct meaning.

#### 4.3.3 Missing values

Some of the records in the dataset contained missing values. We observed 35 missing values found in FY 2018, 68 in FY 2019, and 15 in FY 2021. Most of the missing entries were for the second-level plant description. These missing values were manually treated using the load loss reason in the corresponding year to find the text that signifies the plant area. Where the reason text did not provide explicit guidance, we inferred the value from the previous years by searching for the same term within all the reason columns. Examples of missing values identified are presented in Table 1, together with the corresponding imputed values.

**Table 1:** Missing values with corresponding imputed values.

Missing value	Imputed value
Unit shutdown due to unavailability of ash conveying	Ash conveying
Unit trip	Unit control system
ID fans not coping	ID fans
Dust emission high	Gas cleaning
Mill tripped on PA flow	PA fans
Mill feeder cleaning	Mills and feeders
Boiler metal temperature high	Boiler and boiler tubes
EFP seal water differential pressure low	Feed water and feed pumps
SSC not running	Ash plant and SSC
IP stress or HP stress	Turbine and casing
HP turbine exhaust temperature high	Turbine and steam circuit
Turbine hydraulic oil pump pressure low	Turbine and oil system
Reverse power	Electrical and auxiliaries
Reheater outlet temperature high	Boiler and reheater
Load rejection test	Generator and gen breaker

#### 4.3.4 Duplicates

Next, we cleaned and removed any duplicates that were present in the plant description feature. Most of these duplicates were as a result of irregular spaces such as Draught Group reported as Draught-Group and C&I recorded as C & I. After removing the duplicates and checking for any missing values that could not be treated, 64,279 observations remained.

### 4.4 Explainable multilayer stack ensemble implementation

The implementation of models was done using the Explainable multilayer stack ensemble (EMSE). The base models for the EMSE was developed with six well known machine learning classification models listed in Table 2. With the logistic regression being the first layer model. The EMSE was applied to improve the performance of prediction. The classifier models were imported from scikit-learn [21]. First, the data was imported using the Pandas Python package.

**Table 2:** Machine learning classification models used in the experiment.

Abbreviation	Model
GNB	Gaussian Naive Bayes
LR	Logistic Regression
RFC	Random Forest Classifier
DTC	Decision Tree Classifier
KNN	K-Nearest Neighbors Classifier
XGBoost	X Gradient Booster Classifier
EMSE	Explainable Multilayer Stack Ensemble

#### 4.4.1 Data splitting

Next, we created the feature and target and split the data into training and test sets. Of the 64,279 observations that remained after pre-processing of the data, 75% was allocated to the training set comprising 48,209 observations and 25% to the test set comprising 16,070 observations. There were four variables, namely: X_train and X_test that represent the feature sets for the training and test data, while y_train and y_test represent the target variable sets for the training and test data. The classification load losses for this study consists of two classes, class zero being PCLF and class one being UCLF. The counts for the binary class on the training set was 732 for the negative or zero class (PCLF) and 47,477 for the positive or class one (UCLF). On the other hand, for test set there were 244 counts of the negative or zero class and 15,826 counts of the positive class or class one.

#### 4.4.2 Hyperparameter optimization

Through the Randomized Search with cross-validation we conducted a hyperparameter tuning to identify the optimal values for the specified models. Table 3 presents the optimal hyperparameter values used for each classifier, as identified by the Randomized Search.

**Table 3:** Hyper-parameters from the randomized search.

Classifiers	Hyper-parameters
Gaussian Naive Bayes	Var smoothing = 6.579332246575682e-07
Logistic Regression	C = 3.3333333333333335
	Penalty = l2
Random Forest Classifier	Criterion = entropy
	Max depth = 10
	Min samples leaf = 1
	Min samples split = 6
	N estimators = 245
Decision Tree Classifier	Criterion = gini
	Max depth = 1
	Min samples leaf = 3
	Min samples split = 6
	Splitter = random
K-Nearest Neighbors Classifier	Weights = uniform
	N neighbors = 10
	Leaf size = 30
	Algorithm = ball tree
X Gradient Booster Classifier	Learning rate = 0.5555555555555556
	Loss = exponential
	N estimators = 305

#### 4.4.3 Training

We fitted the optimized classifiers to the training dataset. After that we used the resulting models to predict the test data and thereafter evaluated the performance of each model on the training set.

Since one limitation of scikit-learn is its inability to directly apply machine learning algorithms to categorical columns (Jolly, 2018), it was necessary to encode our target variables into numerical values for scikit-learn to process them effectively. Thus, for this reason, to make the dataset to be compatible with machine learning algorithms, we first consolidated UCLF and OCLF into a single class labeled as UCLF, as both contribute to unplanned outages1. Next, we converted the categorical variables from the target to numeric binary values. Our label of interest is UCLF, therefore, it is our positive case, and we recoded it as 1. Since PCLF is our normal class, we recoded it as 0. Therefore, we ended with one target variable called Classification, containing only two labels: 1 if the load loss is a UCLF or unplanned outage, and 0 if the load loss is a PCLF or planned outage. The features are the remaining variables.

We utilized the LabelEncoder to initialize an integer encoding object and employed the fit_transform method to adapt each categorical variable into a numerical representation.

Given the significant class imbalance, with UCLF having a larger sample size than PCLF, we found it crucial to implement the class weight method in order to prevent the models from being biased toward the majority class and enhances its capability to accurately predict instances from the underrepresented class.

A significant class imbalance between the majority and minority classes can introduce bias in the predictive performance of Machine Learning algorithms, favoring the majority class (Hasanin et al., 2019). Thus, to ensure that the sample population is a true representation of the entire population of load loss classes under investigation and that the target labels are sufficiently represented in both the training and test sets, we employed a stratified random sampling. Stratified random sampling involves dividing the population into groups based on similar characteristics (Cochran, 1946). Subsequently, one element is selected from each group in proportion to its representation in the population to ensure an unbiased estimation. In our research, the two stratified groups are UCLF and PCLF.

With UCLF and PCLF as the target variables, we utilized the supervised machine learning algorithm to train the models for predicting whether the load loss would be a planned outage or unplanned outage. Next, we proceeded to predict the class for the testing set. Following the predictions, we calculated the classification accuracy, representing the percentage of correct predictions. Nonetheless, as accuracy fails to provide insights into the distribution of response values and the specific types of errors made by the classifier, we also employed the confusion matrix for further analysis.

#### 4.4.4 Evaluation

We employed the k-fold validation method, specifically 5-fold cross validation as our model evaluation procedure to estimate how well the models will generalize when exposed to out of sample data in order to choose the best model. Among the various cross-validation techniques, k-fold is widely preferred (Fushiki, 2011; Geisser, 1975) due to its ease of use and effectiveness in assessing the performance of classification models (Marcot and Hanea, 2021; Jung et al., 2020; Tamilarasi and Rani, 2020). In each iteration, a single fold is utilized as testing data while the remaining folds serve as training data (Wayahdi et al., 2020), and this process is repeated until the entire dataset is evaluated (Vanwinckelen and Blockeel, 2012).

In addition to the k-fold method, to quantify the model performance we utilized the accuracy, precision, recall also known as true positive rate or sensitivity, and F1-score. According to Mohammed et al. (2020) accuracy gives more importance to the majority target than the minority target, hence it becomes challenging for a classifier to perform well on the minority target. To address this challenge, we incorporated the F-measure, a combination of precision and recall (Bradley, 1997). The F-measure is widely used as an evaluation metric (Estabrooks and Japkowicz, 2001). Due to the imbalanced nature of our dataset, we utilized the F1 score. The F1 score is a variant of the F-measure, which equally considers precision and recall (He and Ma, 2013). Recall focuses on the model’s ability to identify all relevant positive instances, specifically measuring the proportion of actual positives that were correctly identified, whereas precision underscores the reliability of the model’s positive predictions, focusing on the ratio of predicted positives that are indeed true positives (Bishnoi et al., 2022). In addition, we used the balanced accuracy, Receiver Operating Characteristics (ROC) and Precision-Recall Area Under the Curve (PR-AUC) given that our dataset is highly imbalanced.

To understand the performance of our models, we mapped the actual load losses and predicted load losses in a confusion matrix, which is defined as “a tabular representation commonly employed to describe the performance of classification models or classifiers using a known set of test data.” Given that our dataset comprises two distinct classes: PCLF (0) and UCLF (1), the resulting matrix is a two-by-two. We designated PCLF as a negative class and UCLF as the positive class because the objective is to proactively take measures to reduce UCLF in order to combat plant failures.

We also conducted a feature importance analysis for the best selected model to determine the most relevant features that contributed to the performance of the model. The significance of the features highlights the statistical relevance of each attribute within the dataset in relation to its impact on the model (Belete and Huchaiah, 2022).

#### 4.4.5 Explainability and interpretability

To facilitate meaningful interpretations of the predictive outcomes, we employed the SHAP methodology, which is the approach for XAI focused on ensuring the interpretability of the model’s predictions post-training. SHAP provides the explanations for the decision-making process of the model and highlights the factors influencing its predictions. This approach allows us not only to identify instances of load losses but also to gain insights into how the model formulates its predictions. Such interpretability aids in enhancing our understanding of the model’s performance, fosters trust in the results it produces, and improves the efficacy of decision-making regarding the reduction of UCLF. We performed a sensitivity analysis using the SHAP dependence plot to identify any sources of uncertainty in prediction and to understand the interaction between parameters.

### 4.5 Models

#### 4.5.1 Gaussian Naive Bayes

The Naive Bayes algorithm functions as a probabilistic model employing the principle of the Bayes theorem to predict classes and categories (Jolly, 2018; Bishnoi et al., 2022). It utilizes a classification approach which relies on the assumption of independence among all predictors (Bishnoi et al., 2022) by employing a probability table that is updated through training data, which facilitates the retrieval of class probabilities based on feature values to predict a new observation (Ray, 2019).

#### 4.5.2 Logistic regression

Logistic regression is a model employed in nonlinear relationships when multiple predictors are present (Neeraj and Maurya, 2020) by defining the boundary between distinct classes (Kashmoola et al., 2022).

#### 4.5.3 Random forest

A random forest classifier is made up of an ensemble of decision trees (Neeraj and Maurya, 2020; Kumar et al., 2022), which it repeatedly generate by drawing random samples from the training dataset, and ultimately derives a final decision through a process of majority voting (Bishnoi et al., 2022).

#### 4.5.4 Decision tree

A decision tree is a hierarchical machine learning classifier that resembles an inverted tree, with nodes representing predictor variables, connections illustrating decision paths, and leaf nodes indicating response variables, aiming to predict the target variable’s value through simple decision rules derived from the features (Bishnoi et al., 2022).

#### 4.5.5 KNN

KNN utilizes non-parametric learning methods that do not require assumptions about data distribution; it trains on both positive and negative attributes and classifies new instances by measuring the distance to the nearest training case, or neighbor, with classification determined by the sign of that point (Neeraj and Maurya, 2020).

#### 4.5.6 XGBoost

XGBoost is a boosting-based machine learning classifier primarily employed to minimize bias errors, wherein a sequence of decision trees, referred to as weak learners, is interconnected such that the errors produced by the initial base learners serve as input for subsequent trees, enabling them to learn and correct the identified error patterns (Kumar et al., 2022).

#### 4.5.7 Multilayer stack ensemble

Stacking is a machine learning approach that involves using predictions from models at one level as input features for models at the next level in order to improve the performance of the prediction (Pavlyshenko, 2018). When utilized in isolation, individual models often exhibit certain performance limitations. To address the instability and shortcomings associated with a single model, the ensemble learning approach combines multiple foundational models, thereby producing a more robust predictor (Wang et al., 2024).

## 5 Results

In this section, we present the outcome of the multilayer stack ensemble model implementation, detailing the performance of each model according to the defined performance metrics. Furthermore, we incorporate the findings obtained from the confusion matrix and provide a comparative analysis of each model on the testing dataset. Moreover, we highlight the most relevant features that contributed to the performance of the models.

**Figure 4:**
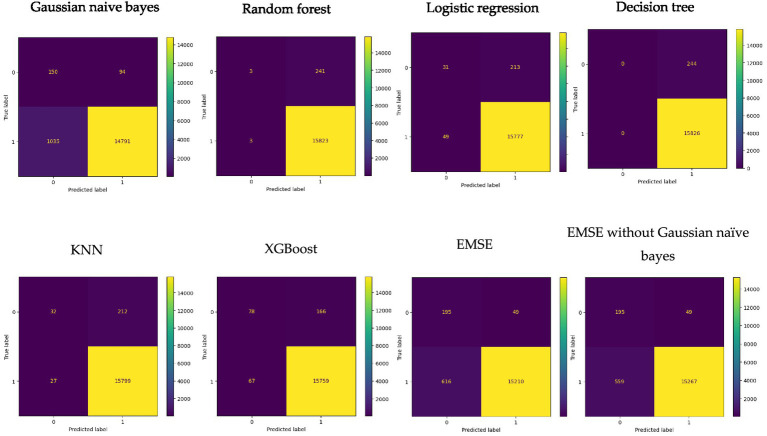
Collective view of matrix for the models.

Figure 4 shows the confusion matrix of the various models. The confusion matrix of the Naive Bayes classifier shows that out of the 16,070 load loss cases, the Gaussian Naive Bayes classifier predicted that 1,185 of load losses will be PCLF and 14,885 will be UCLF. Among the actual load losses, 244 were PCLF, and 15,826 were UCLF. There were 14,791 true positive cases in which the UCLF load losses were correctly predicted as UCLF and 150 true negative cases in which the PCLF load losses were correctly predicted as PCLF. Conversely, there were 94 false positive cases or a type 1 error where PCLF was incorrectly predicted as UCLF, and 1,035 false negative cases or a type 2 error where UCLF was falsely predicted as PCLF.

The confusion matrix of the Random Forest classifier indicates that out of the 16,070 load loss predictions, the Random Forest classifier predicted six load loss instances as PCLF and 16,064 as UCLF. There were 15,823 true positive cases in which the UCLF load losses were correctly predicted as UCLF and three true negative cases in which the PCLF load losses were correctly predicted as PCLF. Conversely, there were 241 load loss cases, which were falsely predicted as UCLF, while, in actual fact, they were PCLF. Furthermore, there were three false negative cases where load losses were incorrectly predicted as PCLF instead of UCLF.

The confusion matrix of the Logistic Regression classifier points that out of the 16,070 load losses, the Logistic Regression classifier predicted 80 load loss instances as PCLF and 15,990 as UCLF. There were 15,777 true positive cases in which the UCLF load losses were correctly predicted as UCLF and 31 true negative cases in which the PCLF load losses were correctly predicted as PCLF. On the contrary, there were 213 load loss cases, which were falsely predicted as UCLF, while, in fact, they were PCLF. Moreover, there were 49 false negative cases of load losses that were incorrectly predicted as PCLF when, in fact, they were UCLF load losses.

The confusion matrix of the Decision Tree classifiers reveals that of the 16,070 load loss predictions, the Decision Tree classifier predicted zero load loss instances as PCLF and 16,070 as UCLF. In actual fact, there were 244 PCLF load loss cases that were falsely predicted as UCLF while on the other hand 15,826 of the UCLF cases were correctly predicted as UCLF.

The confusion matrix of the KNN classifier demonstrates that out of the 16,070 load loss predictions, the KNN classifier predicted 59 load loss instances as PCLF and 16,011 as UCLF. There were 15,799 true positive load loss cases with the correct prediction of UCLF and 32 true negative cases where PCLF load losses were predicted correctly. To the contrary, there were 212 PCLF load losses, which were falsely predicted as UCLF, and 27 false negative cases of UCLF load losses that were incorrectly predicted as PCLF.

The confusion matrix of the XGBoost classifier depicts that out of the 16,070 load loss predictions, the XGBoost classifier predicted 145 load loss instances as PCLF and 15,925 as UCLF. There were 15,759 true positive load loss cases with the correct prediction of UCLF and 78 true negative load loss cases where PCLF load was predicted correctly. On the contrary, there were 166 PCLF load losses, which were falsely predicted as UCLF, and 67 false negative cases of UCLF load losses that were incorrectly predicted as PCLF.

**Figure 5:**
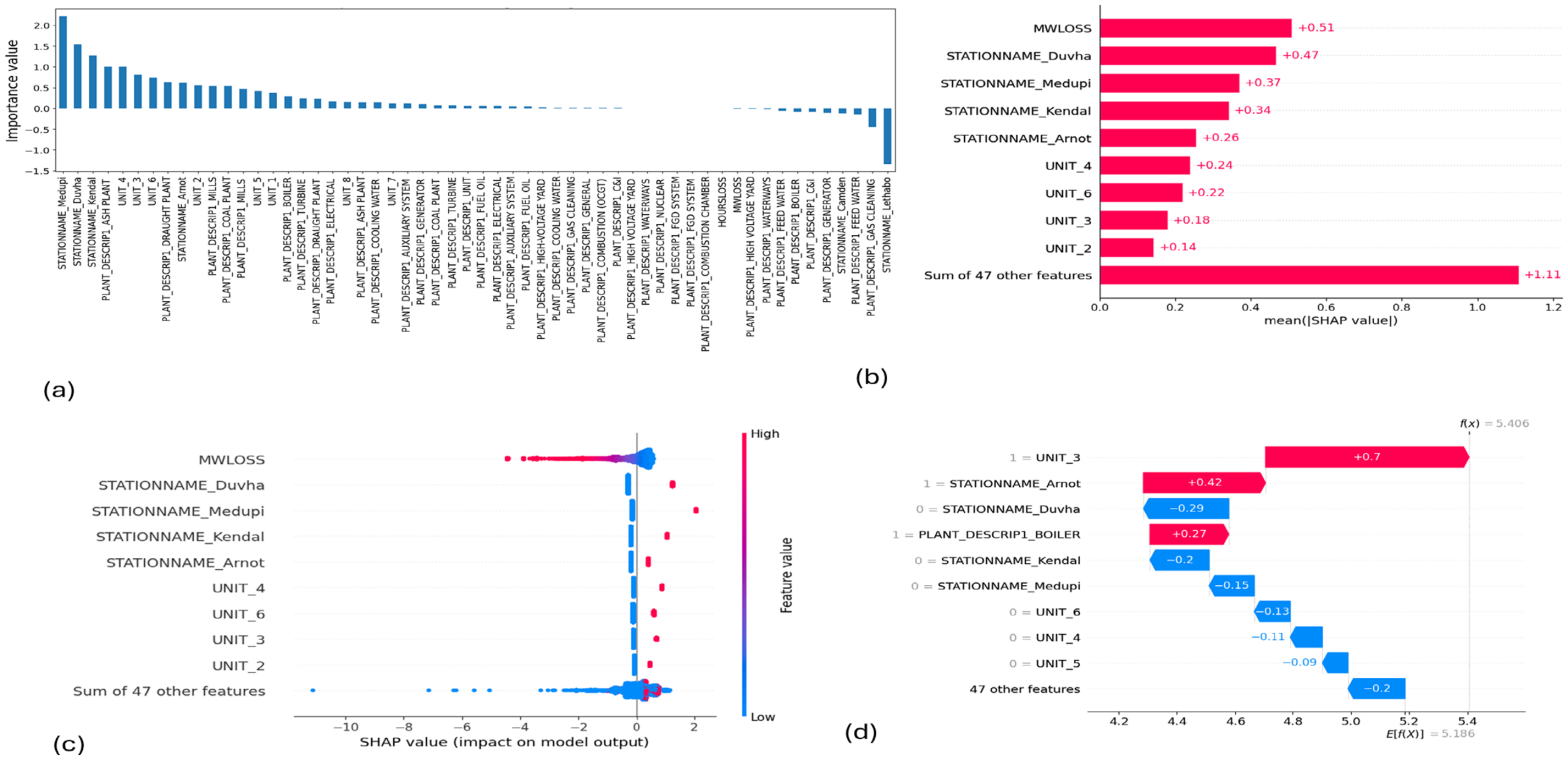
SHAP plots for logistic regression. **(a)** Logistic regression feature importance, **(b)** Logistic regression mean SHAP plot, **(c)** Logistic regression beeswarm plot, **(d)** Logistic regression waterfall plot.

The confusion matrix of the multilayer stack ensemble model highlights that out of the 16,070 load loss predictions, the multilayer stack ensemble classifier predicted 811 load loss instances as PCLF and 15,259 as UCLF. The multilayer stack ensemble classifier correctly predicted 15,210 true positive load loss cases as UCLF and 195 true negative load loss cases as PCLF. On the other hand, there were 49 PCLF load losses, which were incorrectly predicted as UCLF, and 616 false negative cases of UCLF load losses that were incorrectly predicted as PCLF (Figure 5).

In the multilayer stack ensemble without Gaussian Naïve Bayes, out of the 16,070 load loss predictions, the classifier predicted 754 load loss instances as PCLF and 15,316 as UCLF. The classifier correctly predicted 15,267 true positive load loss cases as UCLF and 195 true negative load loss cases as PCLF. On the other hand, there were 49 PCLF load losses, which were falsely predicted as UCLF, and 559 false negative cases of UCLF load losses that were incorrectly predicted as PCLF. With reference to the collective view of the confusion matrix in Figure 4, as well as the individual confusion matrix in Table 4, the Gaussian Bayesian model has the highest number of false negative load loss predictions compared to other models in the stack.

**Table 4:** Overall performance of models.

Model	Correct prediction (%)	F1 score	Precision	Recall	Balanced accuracy	ROC score	PR-AUC	False positive	False negative
Random forest	98.481643	0.992349	0.984998	0.99981	0.506053	0.506053	0.999277	1.50%	0.02%
Logistic regression	98.369633	0.991765	0.986679	0.996904	0.561977	0.561977	0.998399	1.33%	0.30%
Decision tree	98.481643	0.99235	0.984816	1	0.5	0.5	0.994364	1.52%	0%
XGBoost	98.550093	0.992662	0.989576	0.995766	0.657719	0.657719	0.999387	1.03%	0.42
KNN	98.512757	0.992493	0.986759	0.998294	0.564721	0.564721	0.997165	1.32%	0.17%
Gaussian Naïve Bayes	92.974487	0.963238	0.993685	0.934601	0.774678	0.774678	0.99633	0.58%	6.44%
EMSE	95.861854	0.978607	0.996789	0.961077	0.880129	0.880129	0.999201	0.30%	3.83%
EMSE without GNB	96.216553	0.980477	0.996801	0.964678	0.881929	0.881929	0.998997	0.30%	3.48%

Table 4 displays the performance results for each model used in the multilayer stack ensemble. The decision tree achieved the highest recall of one (1) and the lowest false negative of 0 % (0%). The XGBoost has the highest correct prediction, f1-score, and PR-AUC of 98.550093, 0.992662, and 0.999387, respectively. The Gaussian Naïve Bayes has the lowest performance in correct prediction, f1-score, recall, and false negative of 92.974487, 0.963238, 0.934601, and 6.44%, respectively. The stacking showed good performance in precision, balanced accuracy, ROC, and false positives of 0.996789, 0.880129, 0.880129, and 0.30%. On the other hand, the stacking without the Gaussian Naïve Bayes achieved overall improved results of 0.996801 in precision, 0.881929 in balance accuracy, 0.881929 in ROC, and a similar performance of 0.30% in false positives, with a better performance of 3.48% in false negatives compared to the stacking that includes the Gaussian Naïve Bayes.

**Figure 6:**
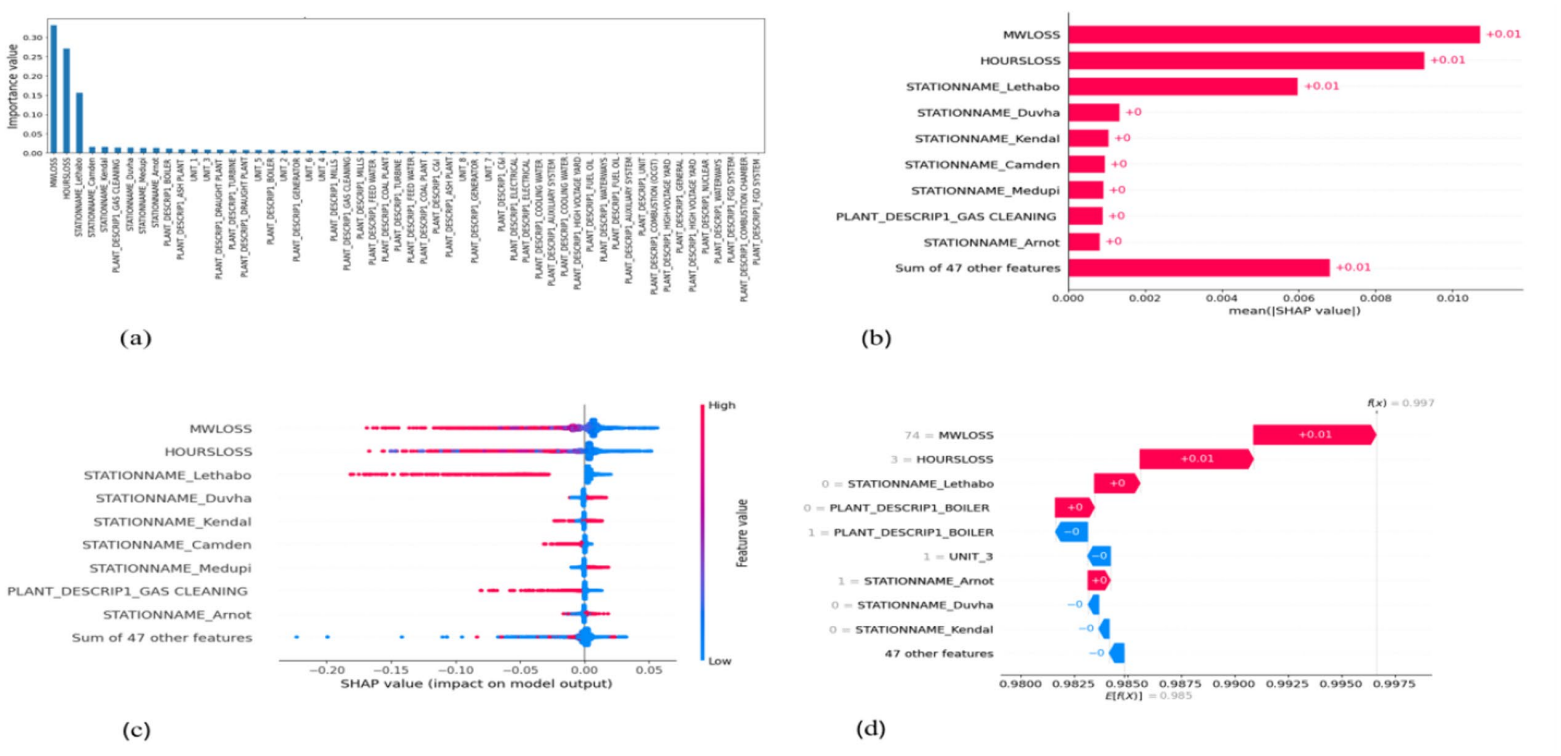
SHAP plots for random regression. **(a)** Random forest feature importance, **(b)** Random forest mean SHAP plot, **(c)** Random forest beeswarm plot, **(d)** Random forest waterfall plot.

**Figure 7:**
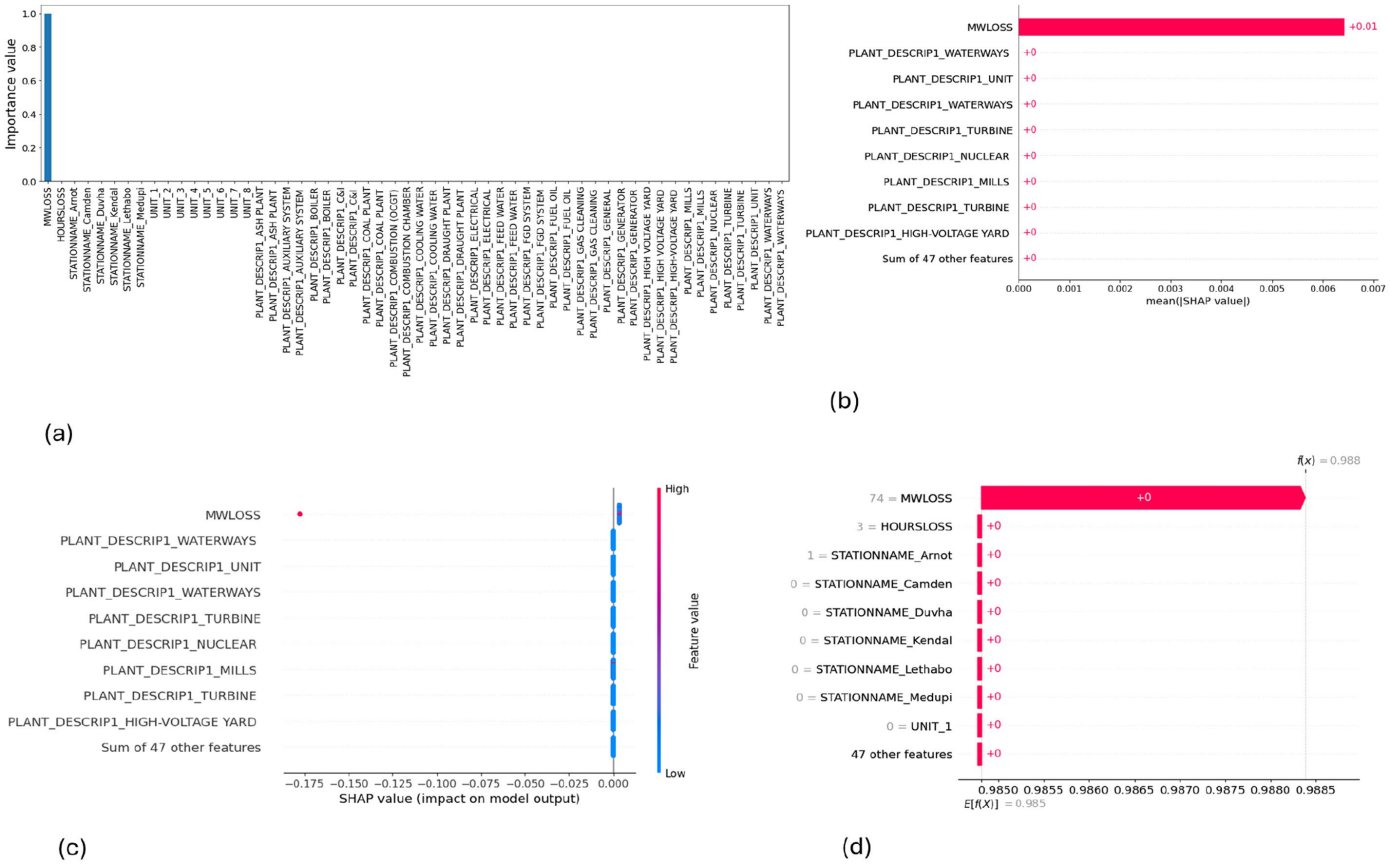
SHAP plots for decision tree. **(a)** Decision tree feature importance, **(b)** Decision tree mean SHAP plot, **(c)** Decision tree beeswarm plot, **(d)** Decision tree waterfall plot.

**Figure 8:**
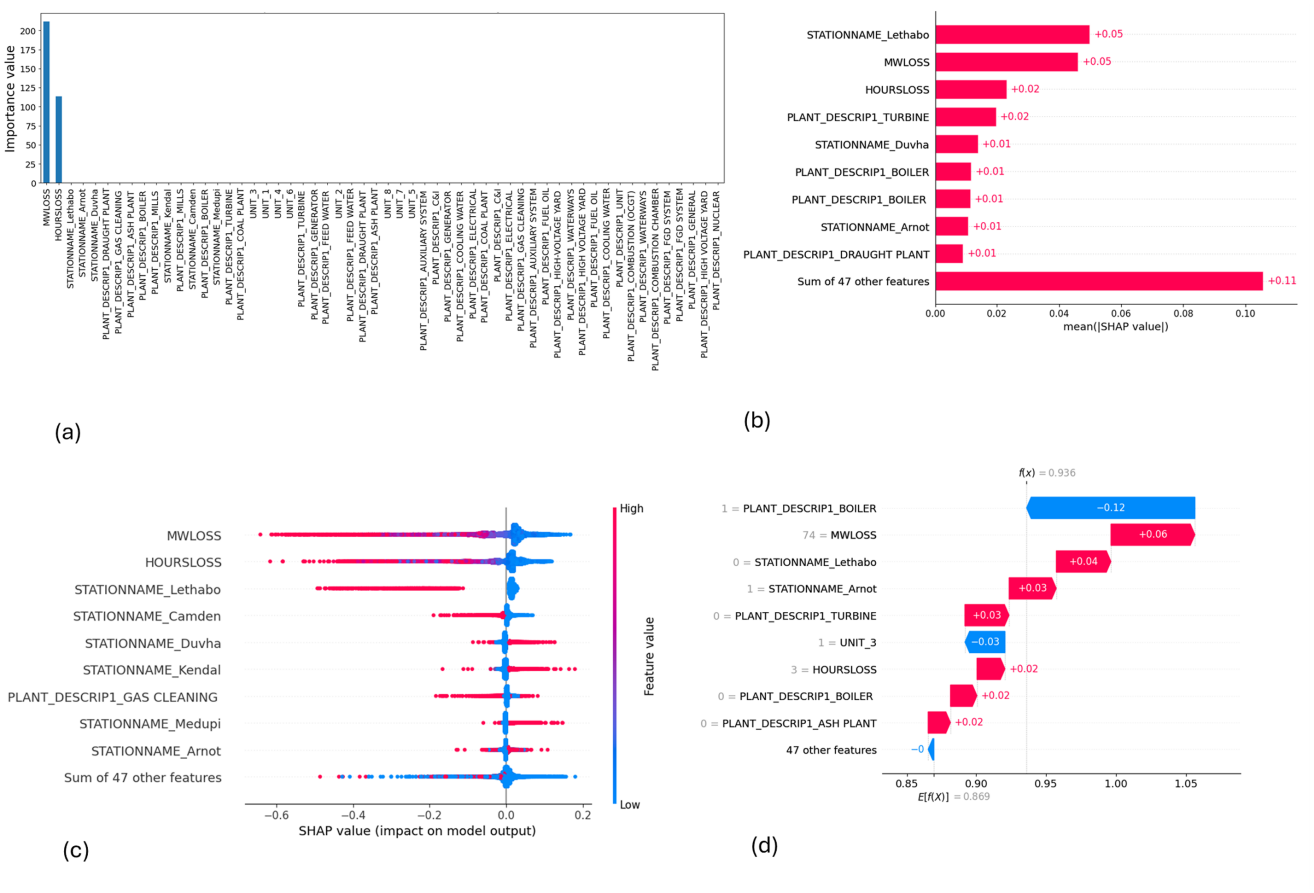
SHAP plots for Gaussian Naive Bayes. **(a)** GNB feature importance, **(b)** GNB mean SHAP plot, **(c)** GNB beeswarm plot, **(d)** GNB waterfall plot.

**Figure 9:**
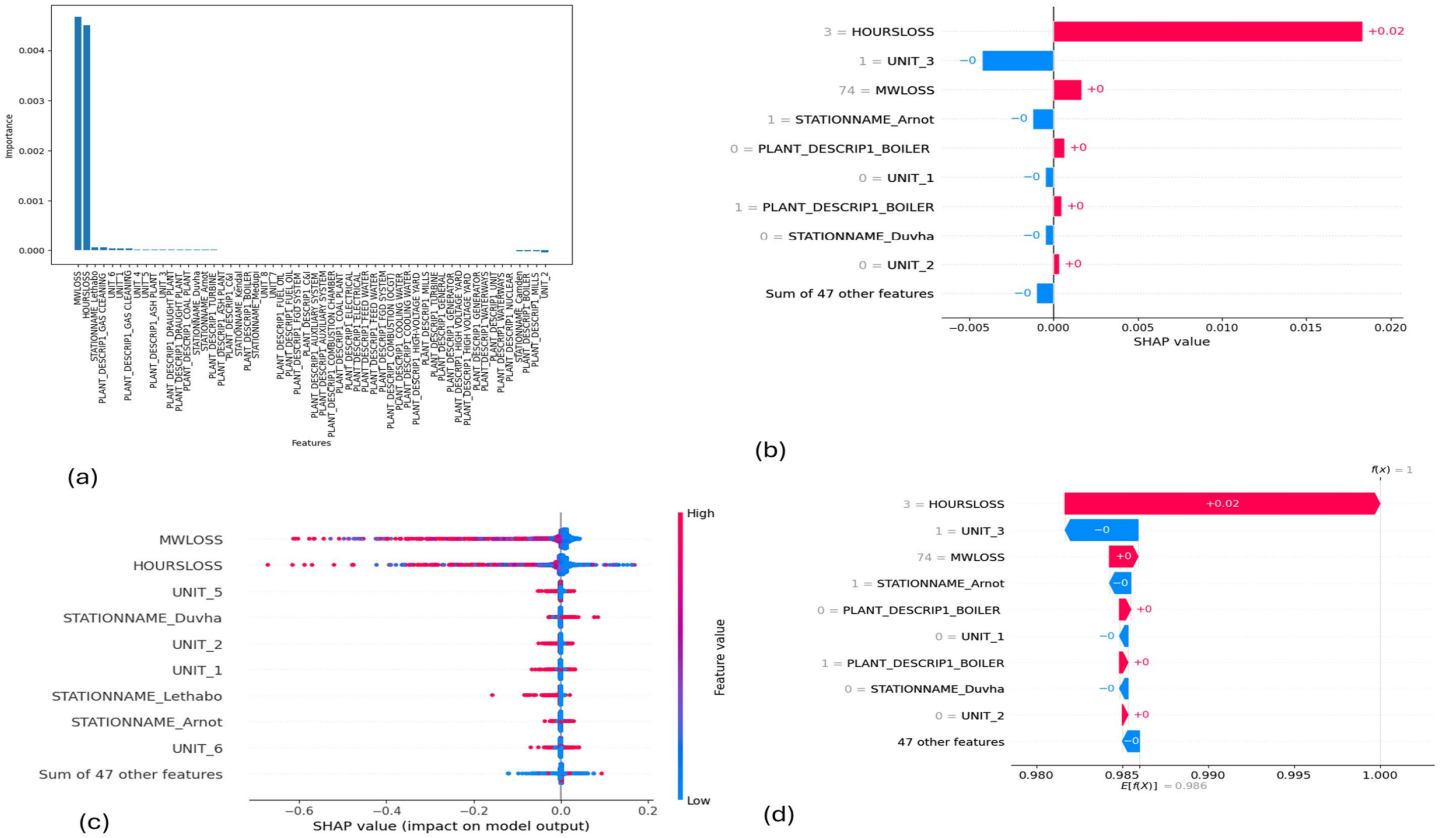
SHAP plots for KNN. **(a)** KNN feature importance, **(b)** KNN mean SHAP plot, **(c)** KNN beeswarm plot, **(d)** KNN waterfall plot.

**Figure 10:**
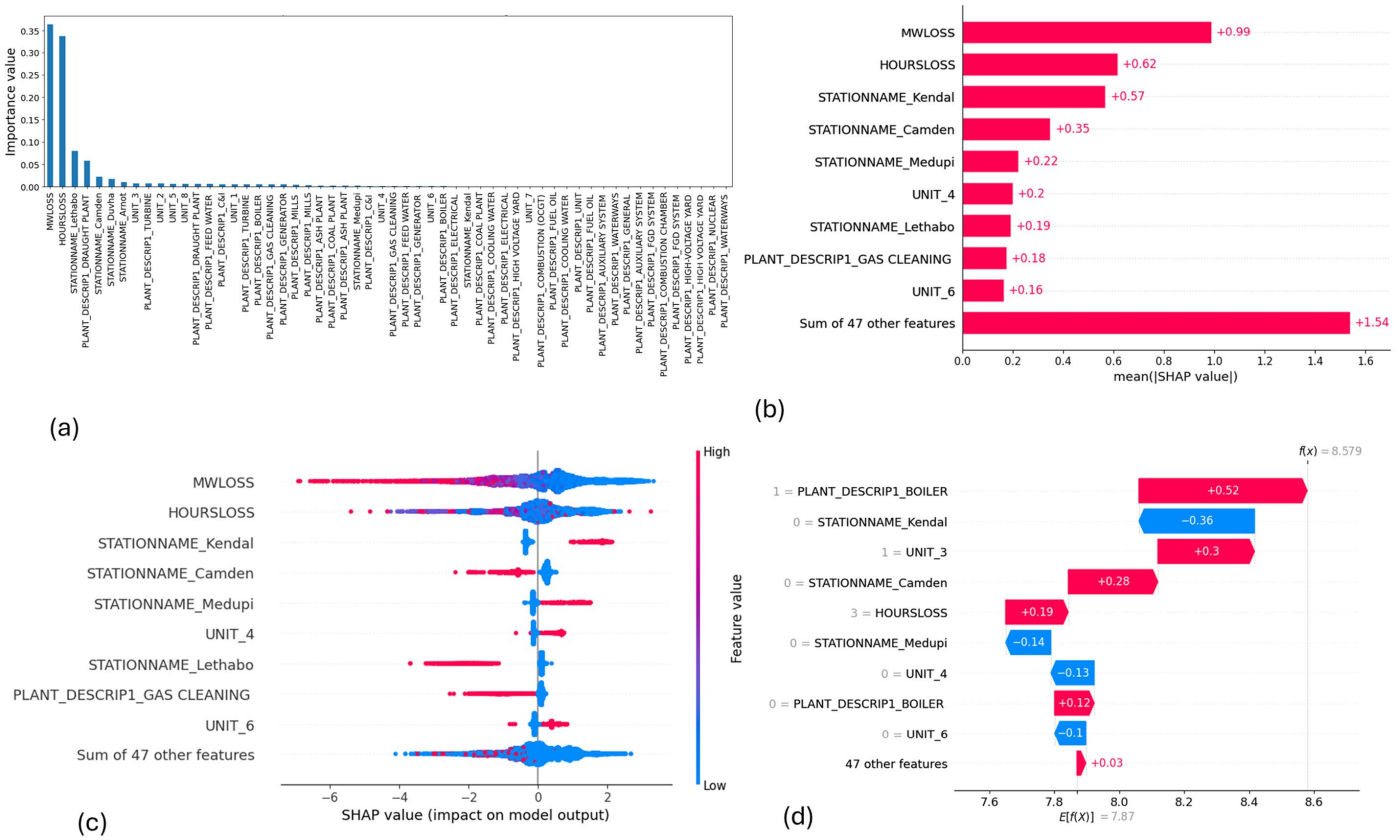
SHAP plots for XGBoost. **(a)** XGBoost feature importance, **(b)** XGBoost mean SHAP plot, **(c)** XGBoost beeswarm plot, **(d)** XGBoost waterfall plot.

**Figure 11:**
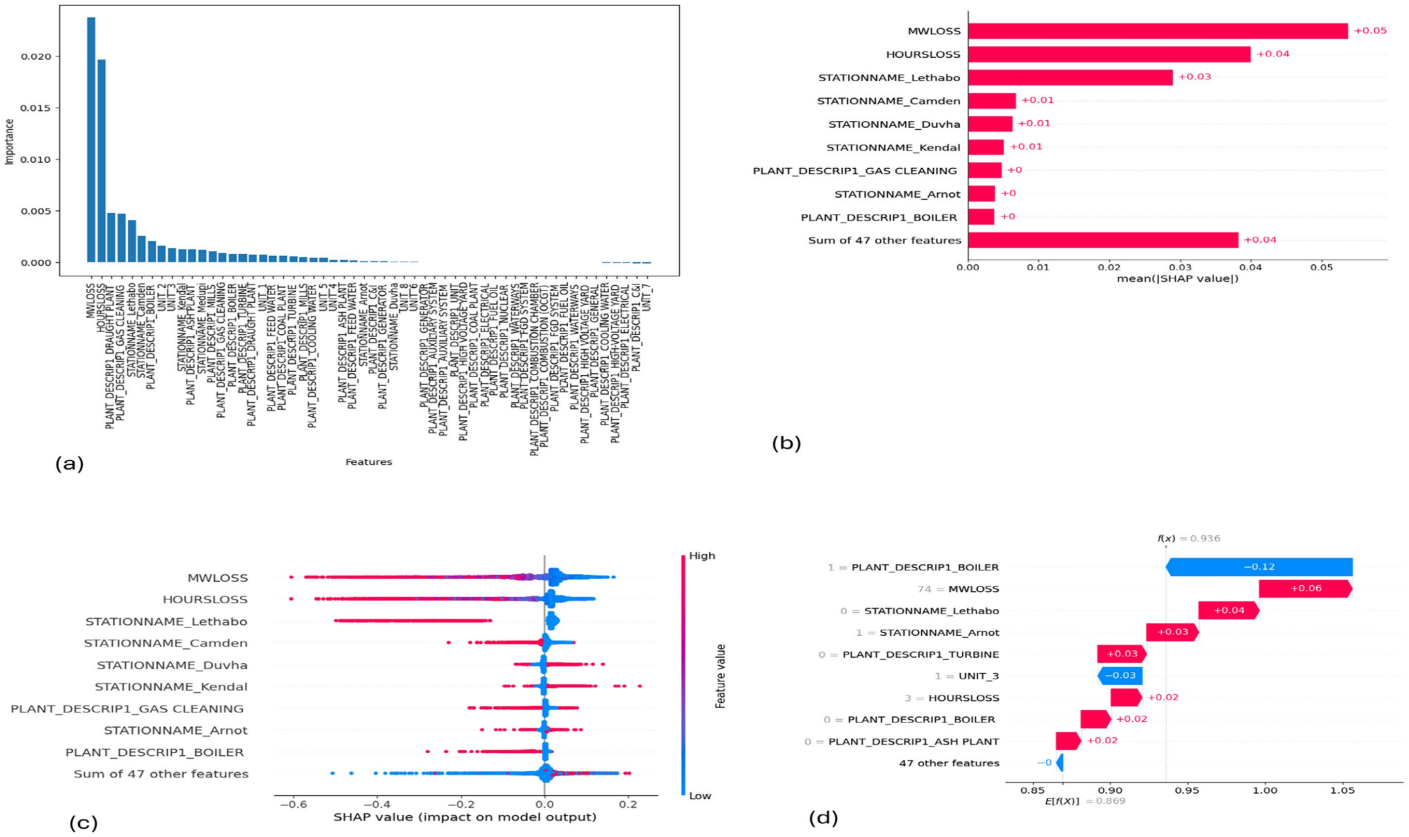
SHAP plots for EMSE. **(a)** EMSE feature importance, **(b)** EMSE mean SHAP plot, **(c)** EMSE beeswarm plot, **(d)** EMSE waterfall plot.

**Figure 12:**
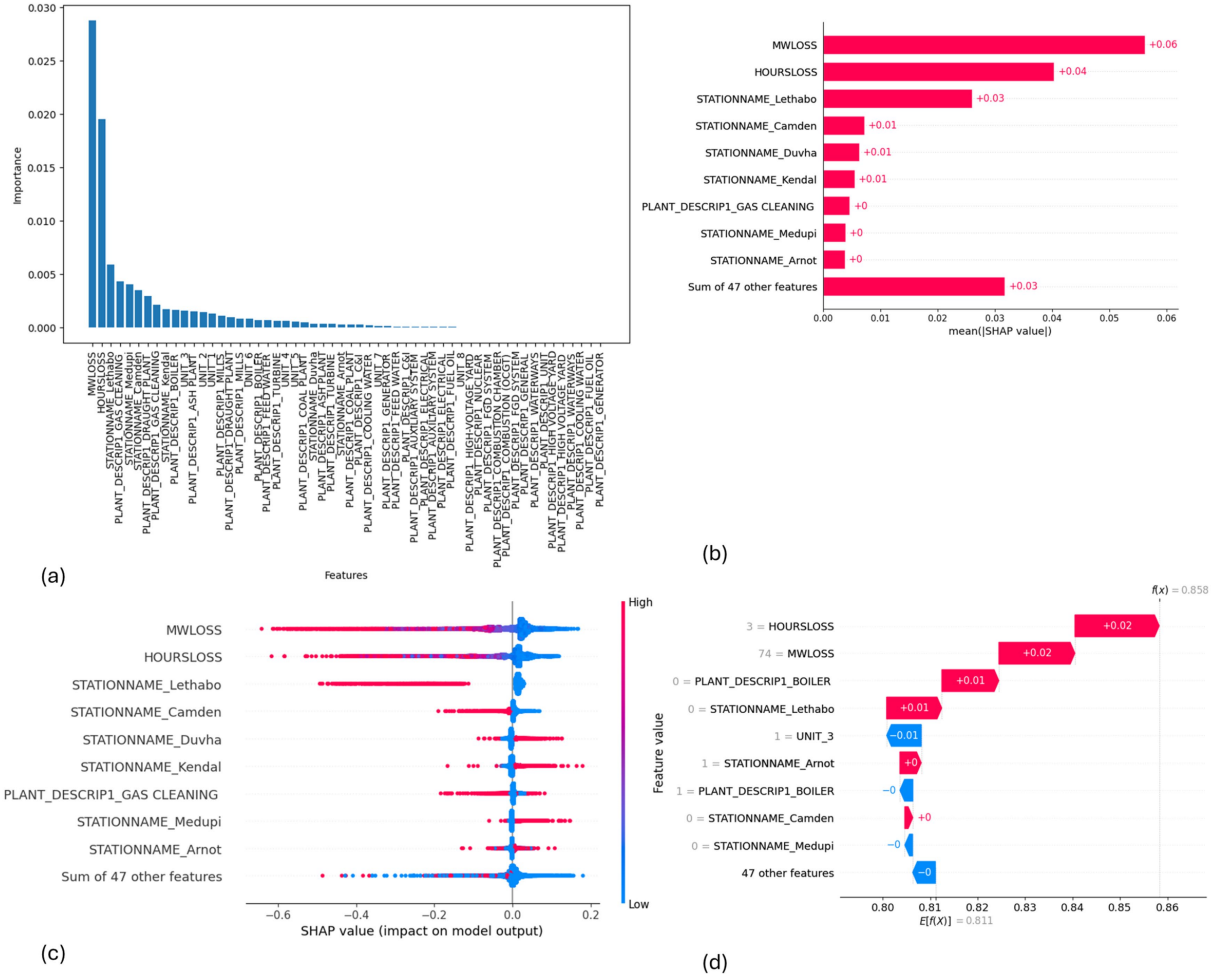
SHAP plots for EMSE without Gaussian Naive Bayes. **(a)** EMSE without GNB feature importance, **(b)** EMSE without GNB mean SHAP plot, **(c)** EMSE without GNB beeswarm plot, **(d)** EMSE without GNB waterfall plot.

**Figure 13:**
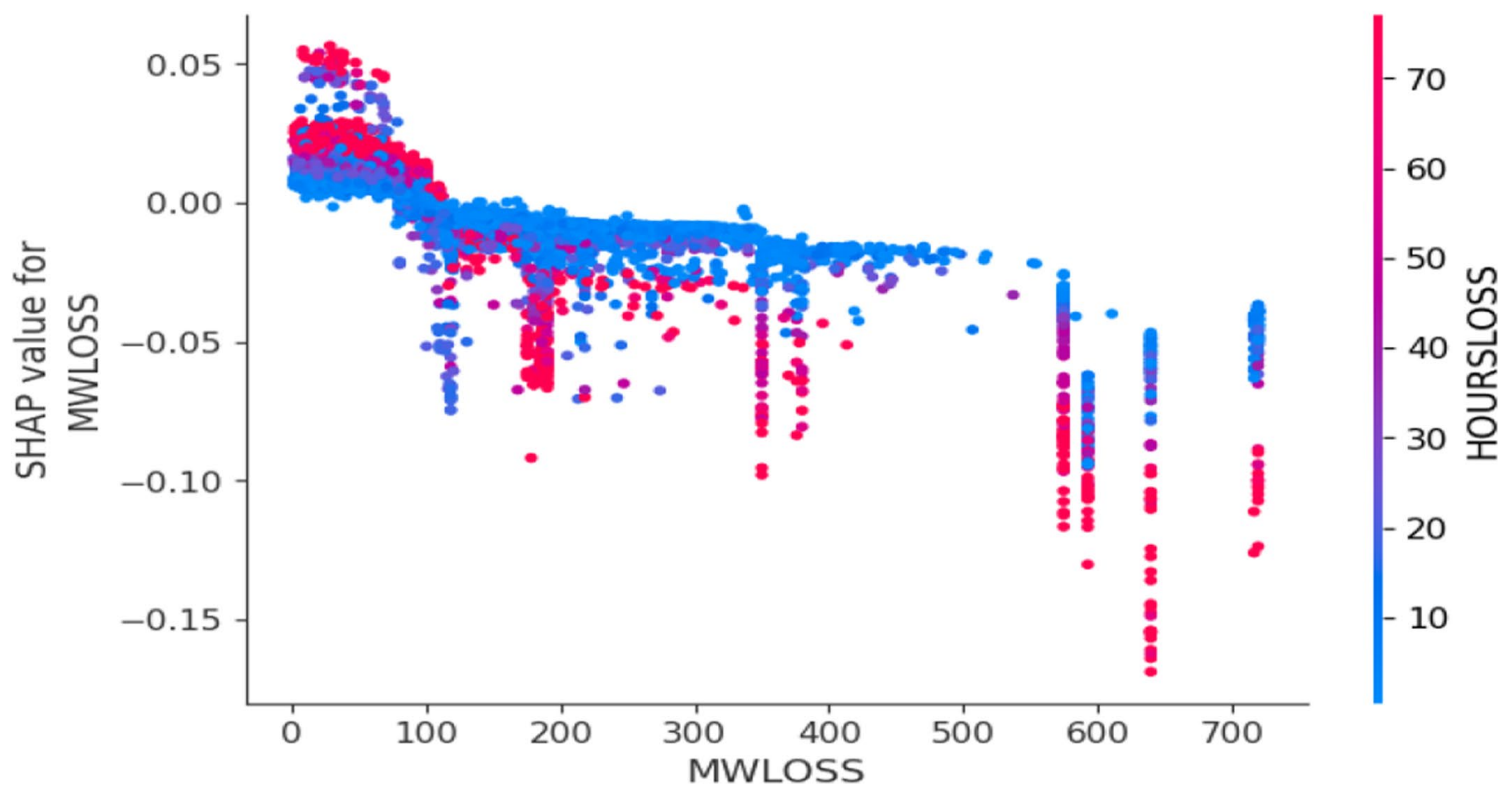
Dependence plot.

MW Loss and Hours Loss are displayed as prominent features in the SHAP plots for the random forest, GNB, KNN, XGBoost, EMSE, and EMSE without GNB in Figures 6–12. In addition, Lethabo power station contributes significantly to the feature importance compared to other power stations. The EMSE plot (b) Figure 12 shows MW Loss, Hours Loss, and Lethabo power station as major contributors to the prediction of load losses. Plot (d) Figure 12 indicates Hours Loss, MW Loss as contributing 0.02 each to the log odds of load losses, and boiler plant and Lethabo power station contributing 0.01 each. In plot (c) of Figure 12, high feature values for Lethabo and Camden power stations show a negative contribution toward load losses observed by the red dots on the left side of the plot, and more so in the gas cleaning plant. On the other hand, high feature values are observed to have a positive impact at Duvha, Kendal, and Medupi power stations indicated by the red dots toward the right side of plot (c) in Figure 12. In Figure 13, we observed that when MW Loss is greater than 150, the impact on the model load loss prediction becomes negative, hence it increases the load loss. Another observation is that when MW Loss is about 650, the SHAP values range between −0.005 and −0.18. Additionally, the impact of the Hours Loss on load loss prediction in relation to MW Loss reveals that the most significant improvement in UCLF occurs when MW Loss is less than 150 and the Hours Loss is below 30.

## 6 Discussion

The results reveal that all the models individually achieved a prediction accuracy exceeding 90%, indicating strong predictive capabilities. However, when comparing standard accuracy with balanced accuracy, we observe a lack of convergence in results. This discrepancy arises from the highly imbalanced nature of our dataset characterized by 244 instances of load losses in the PCLF versus 15,826 in the UCLF cases, which can render accuracy figures somewhat misleading. Therefore, we reviewed the results in conjunction with the balanced accuracy, ROC, and PR-AUC for a more reliable evaluation of model performance. The balanced accuracy metric, in particular, plays a crucial role in providing a more accurate assessment of our models’ performance. Moreover, upon examining the individual models, it was evident that the decision tree exhibits a recall of one (1) and a false negative rate of 0%. At first glance, these results may lead one to conclude that the decision tree model outperforms the other models. However, the balanced accuracy of 0.5 presents a contrasting perspective. These findings suggest that the decision tree model accurately identifies all instances of load losses categorized as UCLF, thus achieving perfect recall, while it erroneously classified all negative cases of load losses as positive. Given that the positive class is considerably more than the negative class, the recall result for the decision tree indicates that the model misclassifies the minority class. Consequently, the balanced accuracy remains low at 0.5 due to the profound imbalance in its predictive capabilities across the respective classes.

In power generation both true positives and true negatives are vital for enhancing plant reliability. Therefore, misclassifying load losses as UCLF, when they are actually PCLF, can result in scheduling unnecessary plant shutdowns, which could be problematic in scenarios of a tight grid where the power supply is insufficient to meet demand. By the same notion, it remains pertinent to address false negatives, where the model incorrectly classifies UCLF load losses as planned load losses as it is the case with the Gaussian Naive Bayes, which produced a high rate of false negatives. High false negative rates could lead to no intervention under the wrong impression that the load losses are PCLF while, in actual fact, they are UCLF losses. Thus, misclassifying a load loss as PCLF when it is UCLF can lead to missed opportunities for essential maintenance, potentially resulting in plant failures and undermining the plant’s reliability. Hence, the further experimentation with the ensemble led to the exclusion of the Gaussian Naïve Bayes model from the multilayer stack ensemble since it produced a high rate of false negatives to avoid its distorted influence on the overall performance of the multilayer stack ensemble model.

In addition, the analysis indicates that the EMSE without the Gaussian Naïve Bayes model offers notable advantages, as evidenced by improved performance of 0.996801 precision, 0.881929 balanced accuracy, and 0.881929 ROC. These improvements surpass the performance of both the individual models and the multilayer stack ensemble model with the Gaussian Naive Bayes. Additionally, although the false negative rates remained consistent with those of the multilayer stack ensemble model, there was a slight improvement in recall, highlighting the efficacy of this modified multilayer stack ensemble model. Overall, the results suggest a promising direction for optimizing model performance using the multilayer stack ensemble model by selectively excluding a model that negatively influences the performance of the multilayer stack ensemble model, thus providing a better-performing multilayer machine learning ensemble.

In light of the above, the overall performance evaluation of our models demonstrates that the EMSE without the Gaussian Naïve Bayes produces improved results, thus making it the ideal model to utilize to support load loss management in power stations. Therefore, based on the evidence of this study, we propose the use of the EMSE without the Gaussian Naïve Bayes for the prediction classification of load losses in power stations, as it produced improved classification performance in line with the objective of this study. In addition, balanced accuracy, ROC, and PR-AUC provide a more reliable evaluation of model performance compared to the standard evaluation metrics such as the accuracy, precision, and recall, when handling a highly imbalanced dataset. Therefore, with the excellent performance of the EMSE presented in this study, we contribute to the management of load losses by offering practical implications as a proactive plant reliability triage for prioritizing which plants to include in the planned outage schedule, enabling a proactive approach that can significantly enhance decision-making and empower the electricity utilities to take timely action to reduce the UCLF and improve overall plant reliability.

From the SHAP plots, higher values of ‘MW Loss and Hours Loss’ features correlate with increased load losses. We conclude that the power utility should focus on reducing these values to mitigate the risk of unplanned outages. Moreover, the high feature values at Lethabo power station and Camden power station, which negatively impact load losses, imply that they contribute significantly toward UCLF. From this analysis, we can conclude that if the power utility wants to curb load losses and reduce unplanned outages, it should prioritize Lethabo power station for planned outages, followed by Camden power station. The area to focus on is the gas cleaning plant, as it is seen as a high-value feature on the left side of the plot, indicating a negative impact. On the other hand, the high feature values with a positive impact at Duvha, Kendal, and Medupi power stations demonstrate PCLF load losses, proving that these stations undertook planned maintenance. Moreover, the dependence plot showing the interaction effect between the MW Loss and Hours Loss reveals that to reduce UCLF, the power stations should maintain MW Loss below 150, schedule planned outages fast to lessen the duration of downtime. Overall, the dependence plots shows that our model is not sensitive to changes in MW Loss or Hours Loss, consistent with all the SHAP plots, the blue dots indicates that low values of MW Loss or Hours Loss, have a positive impact on the load losses, meaning that the lower these values are, the lesser the UCLF will be.

From the above analysis, we have demonstrated how an EMSE without GNB has produced improved results. From the SHAP plots we were able to identify where these load losses are most prominent, thereby, contributing to supporting the power utility in making informed decisions about planned outages for plant areas that require the most urgent attention to reduce UCLF load losses, which have a negative impact to the reliability of electricity generation, backed by empirical research.

## 7 Conclusion

This study addresses the pressing plant reliability problem of reduced plant availability due to load losses, especially those classified as UCLF, faced by many power stations.

Identification of load losses prior to their occurrence is crucial for timely intervention to reduce unplanned outages, thereby enhancing plant reliability and increasing the EAF. Consequently, in our study, we applied machine learning methods to predict load losses in power stations. The study introduced the EMSE model which produced excellent performance.

First, the EMSE consisting of Logistic Regression, Gaussian Naïve Bayes, Decision Tree, Random Forest, KNN, and XGBoost was applied to classify load losses dataset from power stations.

Second, experimentation with the models on the first and second layers of the EMSE, along with evaluation, facilitated a comparative analysis and ultimately led to the identification of the Gaussian Naïve Bayes model as producing skewed results. After excluding it from the stack, the performance of the resulting EMSE improved. The EMSE without the Gaussian Naïve Bayes model achieved improved performance with a prediction accuracy of 96%, a precision rate of 0.996801, and a balanced accuracy of 0.881929.

Third, XAI was integrated into the models to provide transparency and explanations for the model’s decision-making process using a well-known XAI technique called SHAP, pointing toward Lethabo power station as significantly contributing to UCLF, suggesting that in order to reduce load losses, it should be prioritized for a planned outage.

Fourth, class weights are employed to address the issue of a highly imbalanced dataset. Furthermore, the study advocates for the use of balanced accuracy, ROC, and PR-AUC as evaluation metrics to diminish the disproportionate influence of the majority class on predictive outcomes. The findings of our research provide empirical evidence that underscores the validity and applicability of machine learning methodologies. In addition, the study contributes to the management of load losses by offering practical implications as a proactive plant reliability triage. This practical approach can be useful in prioritizing which plants to include in the planned outage schedule, enabling a proactive approach that can significantly enhance decision-making and empower the electricity utilities to take timely action to reduce the UCLF and improve overall plant reliability.

The study has three limitations. First, our models were trained and tested with data from fossil power stations. However, this opens exciting possibilities for future work to expand the study to other power generation regimes. Given the unique characteristics of plant mix, and the operational differences, we recommend future research to explore the prediction classification of load losses to other power stations such as nuclear, which have load loss features that may require distinct model feature engineering strategies due to its complex plant systems such as the nuclear reactors. Second, since our EMSE is based on Gaussian Naïve Bayes, Logistic Regression, Decision Tree, Random Forest, KNN, and XGBoost, future research could explore the development of ensembles that include other classification models such as LSTM or transformers and Neural Networks, to address long sequence data and non-linear relationships. Third, our study concentrated solely on a classification task, therefore, since the load loss events occurs within time stamps, future research can explore a regression task by using techniques such as timeseries. The research implications presented in this study transcend academic boundaries, extending into practical applications that hold relevance for industry. Therefore, the findings of our study can be deployed to electricity utilities and leveraged to devise a digital framework for the decision-making process in outage planning, ultimately optimizing the reliability of power stations.

## Data Availability

The data analyzed in this study is subject to the following licenses/restrictions: permission to receive data can be obtained from the data owner. Requests to access these datasets should be directed to Eskom, DhaverK@eskom.co.za.

## Footnotes

^1^ https://www.eskom.co.za/wp-content/uploads/2023/10/Eskom_integrated_report_2023.pdf

## References

[ref1] Abaas M. Lee R. A. Singh P.. Long short-term memory customer-centric power outage prediction models for weather-related power outages. 2022 IEEE Green Energy and Smart System Systems (IGESSC), (2022). 1–6.

[ref2] Aghahadi M. Bosisio A. Merlo M. Berizzi A. Pegoiani A. Forciniti S. (2024). Digitalization processes in distribution grids: a comprehensive review of strategies and challenges. Appl. Sci. 14:4528. doi: 10.3390/app14114528

[ref3] Belete D. M. Huchaiah M. D. (2022). Grid search in hyperparameter optimization of machine learning models for prediction of HIV/AIDS test results. Int. J. Comput. Appl. 44, 875–886. doi: 10.1080/1206212X.2021.1974663

[ref4] Bishnoi S. Al-Ansari N. Khan M. Heddam S. Malik A. (2022). Classification of cotton genotypes with mixed continuous and categorical variables: application of machine learning models. Sustainability 14:13685. doi: 10.3390/su142013685

[ref5] Bradley A. P. (1997). The use of the area under the ROC curve in the evaluation of machine learning algorithms. Pattern Recogn. 30, 1145–1159. doi: 10.1016/S0031-3203(96)00142-2

[ref6] Brossa Dachs N. (2018) Machine learning in classification of latex gloves

[ref7] Bush B. Chen Y. Ofori-Boateng D. Gel Y. R. (2021). Topological machine learning methods for power system responses to contingencies. Proc. AAAI Conf. Artificial Intelligence 35, 15262–15269. doi: 10.1609/aaai.v35i17.17791

[ref8] Čačković V. Popović Ž.. Role of Data Analytics in Utilities Transformation. 2016 39th International Convention on Information and Communication Technology, Electronics and Microelectronics (MIPRO), (2016). 518–523.

[ref9] Chadaga S. P. (2023). Power failure Cascade prediction using machine learning. United States: Massachusetts Institute of Technology.

[ref10] Chakole J. B. Kolhe M. S. Mahapurush G. D. Yadav A. Kurhekar M. P. (2021). A Q-learning agent for automated trading in equity stock markets. Expert Syst. Appl. 163:113761. doi: 10.1016/j.eswa.2020.113761

[ref11] Cochran W. G. (1946). Relative accuracy of systematic and stratified random samples for a certain class of populations. Ann. Math. Stat. 17, 164–177. doi: 10.1214/aoms/1177730978

[ref12] Dalal N. Mølna M. Herrem M. Røen M. Gundersen O. E. (2021). Day-ahead forecasting of losses in the distribution network. AI Mag. 42, 38–49. doi: 10.1609/aimag.v42i2.15097

[ref13] Estabrooks A. Japkowicz N. A mixture-of-experts framework for learning from imbalanced data sets. International Symposium on Intelligent Data Analysis, (2001). 34–43.

[ref14] Fushiki T. (2011). Estimation of prediction error by using K-fold cross-validation. Stat. Comput. 21, 137–146. doi: 10.1007/s11222-009-9153-8

[ref15] García S. Ramírez-Gallego S. Luengo J. Benítez J. M. Herrera F. (2016). Big data preprocessing: methods and prospects. Big Data Anal. 1, 1–22. doi: 10.1186/s41044-016-0014-0

[ref16] Geisser S. (1975). The predictive sample reuse method with applications. J. Am. Stat. Assoc. 70, 320–328. doi: 10.1080/01621459.1975.10479865

[ref17] Gils H. C. Bothor S. Genoese M. Cao K. K. (2018). Future security of power supply in Germany—the role of stochastic power plant outages and intermittent generation. Int. J. Energy Res. 42, 1894–1913. doi: 10.1002/er.3957

[ref18] Grando F. L. Lazzaretti A. E. Moreto M. Lopes H. S. Fault classification in power distribution systems using PMU data and machine learning. 2019 20th International Conference on Intelligent System Application to Power Systems (ISAP), (2019). 1–6.

[ref19] Hagberg J. Sundstrom M. Egels-Zandén N. (2016). The digitalization of retailing: an exploratory framework. Int. J. Retail Distrib. Manag. 44, 694–712. doi: 10.1108/IJRDM-09-2015-0140

[ref20] Han-Min L. Chae-Joo M. (2022). Regional analysis of load loss in power distribution lines based on Smartgrid big data. J. Korea Institute Electron. Commun. Sci. 17, 1013–1024. doi: 10.13067/JKIECS.2022.17.6.1013

[ref21] Hasanin T. Khoshgoftaar T. M. Leevy J. L. Bauder R. A. (2019). Severely imbalanced big data challenges: investigating data sampling approaches. J. Big Data 6, 1–25. doi: 10.1186/s40537-019-0274-4

[ref22] He H. Ma Y. (2013). Imbalanced learning: Foundations, algorithms, and applications.

[ref23] Hou H. Wang Z. Chen Y. Wang Q. Zhao B. Zhang Q.. (2023). Multi-stage hybrid energy management strategy for reducing energy abandonment and load losses among multiple microgrids. Int. J. Electr. Power Energy Syst. 148:108773. doi: 10.1016/j.ijepes.2022.108773

[ref24] Hustoft J. Weber B. (2019). The impact of digital transformation on the electric power industry: An explorative study of the largest norwegian distribution system operators.

[ref25] Jackson J. (2002). Data mining; a conceptual overview. Commun. Assoc. Inf. Syst. 8:19. doi: 10.17705/1CAIS.00819

[ref26] Jolly K. (2018). Machine learning with scikit-learn quick start guide: Classification, regression, and clustering techniques in Python: Packt Publishing Ltd.

[ref27] Jung K. Bae D.-H. Um M.-J. Kim S. Jeon S. Park D. (2020). Evaluation of nitrate load estimations using neural networks and canonical correlation analysis with k-fold cross-validation. Sustainability 12:400. doi: 10.3390/su12010400

[ref28] Kashmoola M. A. Ahmed M. K. Alsaleem N. Y. A. (2022). Network traffic prediction based on boosting learning. Iraqi J. Sci., 4047–4056. doi: 10.24996/ijs.2022.63.9.33

[ref29] Kraus S. Jones P. Kailer N. Weinmann A. Chaparro-Banegas N. Roig-Tierno N. (2021). Digital transformation: an overview of the current state of the art of research. SAGE Open 11:21582440211047576. doi: 10.1177/21582440211047576

[ref30] Kumar R. S. Ram S. S. Jayakar S. A. Kumar T. S. (2022). Failure prediction of turbines using machine learning algorithms. Mater Today Proc 66, 1175–1182. doi: 10.1016/j.matpr.2022.04.984

[ref31] Li S. Ding T. Jia W. Huang C. Catalão J. P. Li F. (2021). A machine learning-based vulnerability analysis for cascading failures of integrated power-gas systems. IEEE Trans. Power Syst. 37, 2259–2270. doi: 10.1109/TPWRS.2021.3119237

[ref32] Li Y. (2017). Deep reinforcement learning: An overview. arXiv preprint. doi: 10.48550/arXiv.1701.07274

[ref33] Li Y. Liu P. Wang Z. (2022). Stock trading strategies based on deep reinforcement learning. Sci. Program. 2022:4698656. doi: 10.1155/2022/4698656

[ref34] Mahgoub M. O. (2021). Real-time prediction of cascading failures in power systems. Univ. Saskatchewan. doi: 10.1109/EPEC48502.2020.9320125

[ref35] Mahgoub M. O. Mazhari S. M. Chung C. Faried S. O.. A prediction interval based cascading failure prediction model for power systems. 2020 IEEE Electric Power and Energy Conference (EPEC), (2020). 1–6.

[ref36] Majzoobi A. Mahoor M. Khodaei A. Machine learning applications in estimating transformer loss of life. 2017 IEEE Power & Energy Society General Meeting, (2017). 1–5.

[ref37] Management M. I. O. T. S. O. Brown S. (2021). Machine learning, explained. MIT: Sloan management.

[ref38] Marcot B. G. Hanea A. M. (2021). What is an optimal value of k in k-fold cross-validation in discrete Bayesian network analysis? Comput. Stat. 36, 2009–2031. doi: 10.1007/s00180-020-00999-9

[ref39] Marsland S. (2011). Machine learning: An algorithmic perspective. New York: Chapman and Hall/CRC.

[ref40] Marx A. Rousseau P. Laubscher R. (2021). Development and validation of a robust integrated thermal power plant model for load loss analysis and identification. MATEC Web Conf. 347. doi: 10.1051/matecconf/202134700011

[ref41] Meng T. L. Khushi M. (2019). Reinforcement learning in financial markets. Data 4:110. doi: 10.3390/data4030110

[ref42] Mitchell T. M. Mitchell T. M. (1997). Machine learning. New York: McGraw-hill.

[ref43] Mohammed R. Rawashdeh J. Abdullah M.. Machine learning with oversampling and undersampling techniques: overview study and experimental results. 2020 11th international conference on information and communication systems (ICICS), (2020). 243–248.

[ref44] Morakanyane R. Grace A. A. O'reilly P. (2017) Conceptualizing digital transformation in business organizations: A systematic review of literature

[ref45] Nakas G. A. Dirik A. Papadopoulos P. N. Matavalam A. R. R. Paul O. Tzelepis D. (2023). Online identification of cascading events in power systems with renewable generation using measurement data and machine learning. IEEE Access 11, 72343–72356. doi: 10.1109/ACCESS.2023.3294472

[ref46] Nazari-Heris M. Asadi S. Mohammadi-Ivatloo B. Abdar M. Jebelli H. Sadat-Mohammadi M. (2021). Application of machine learning and deep learning methods to power system problems: Springer.

[ref47] Neeraj K. N. Maurya V. (2020). A review on machine learning (feature selection, classification and clustering) approaches of big data mining in different area of research. J. Crit. Rev. 7, 2610–2626. doi: 10.31838/jcr.07.19.322

[ref48] Nist B. (2015) NIST Big Data Interoperability Framework: Volume 1, Definitions. NIST Big Data Public Working Group Gaithersburg, MD, USA

[ref49] Nti I. K. Nyarko-Boateng O. Aning J. (2021). Performance of machine learning algorithms with different K values in K-fold crossvalidation. Int. J. Inform. Technol. Computer Sci. 13, 61–71. doi: 10.5815/ijitcs.2021.06.05

[ref50] O’donnell J. Su W. (2023). A stochastic load forecasting approach to prevent transformer failures and power quality issues amid the evolving electrical demands facing utilities. Energies 16:7251. doi: 10.3390/en16217251

[ref51] Ogieva F. Ike A. Anyaeji C. (2015). Egbin power station generator availability and unit performance studies. Int. J. Phys. Sci. 10, 155–172. doi: 10.5897/IJPS2014.4226

[ref52] Omogoye S. O. Folly K. A. Awodele K. O. (2023). A comparative study between Bayesian network and hybrid statistical predictive models for proactive power system network resilience enhancement operational planning. IEEE Access 11, 41281–41302. doi: 10.1109/ACCESS.2023.3263490

[ref53] Pavlyshenko B.. Using stacking approaches for machine learning models. 2018 IEEE second international conference on data stream mining & processing (DSMP), (2018). 255–258.

[ref54] Pendharkar P. C. Cusatis P. (2018). Trading financial indices with reinforcement learning agents. Expert Syst. Appl. 103, 1–13. doi: 10.1016/j.eswa.2018.02.032

[ref55] Plevris V. Solorzano G. Bakas N. P. Ben Seghier M. E. A.. Investigation of performance metrics in regression analysis and machine learning-based prediction models. 8th European Congress on Computational Methods in Applied Sciences and Engineering (ECCOMAS Congress 2022), (2022).

[ref56] Powertech V. Booklet 3, fundamentals and systematics of availability for thermal power plants. (2008).

[ref57] Ray S.. A quick review of machine learning algorithms. 2019 International conference on machine learning, big data, cloud and parallel computing (COMITCon), (2019). 35–39.

[ref58] Ruiz-Chavez Z. Salvador-Meneses J. Garcia-Rodriguez J. Machine learning methods based preprocessing to improve categorical data classification. Intelligent Data Engineering and Automated Learning–IDEAL 2018: 19th International Conference, Madrid, Spain, November 21–23, 2018, Proceedings, Part I 19, (2018). 297–304.

[ref59] Sahu S. K. Mokhade A. Bokde N. D. (2023). An overview of machine learning, deep learning, and reinforcement learning-based techniques in quantitative finance: recent progress and challenges. Appl. Sci. 13:1956. doi: 10.3390/app13031956

[ref60] Samuel A. L. (2000). Some studies in machine learning using the game of checkers. IBM J. Res. Dev. 44, 206–226. doi: 10.1147/rd.441.0206

[ref61] Schwertner K. (2017). Digital transformation of business. Trakia Journal of Science 15, 388–393. doi: 10.15547/tjs.2017.s.01.065

[ref62] Sharifzadeh A. Ameli M. T. Azad S. (2021). Power system challenges and issues. Application of Machine Learning and Deep Learning Methods to Power System Problems, 1–17.

[ref63] Shuvo S. S. Yilmaz Y.. Predictive maintenance for increasing EV charging load in distribution power system. 2020 IEEE International Conference on Communications, Control, and Computing Technologies for Smart Grids (Smart Grid Comm), (2020). 1–6.

[ref64] Shuvro R. A. Das P. Hayat M. M. Talukder M.. Predicting cascading failures in power grids using machine learning algorithms. 2019 North American Power Symposium (NAPS), (2019). 1–6.

[ref65] Tamilarasi P. Rani R. U.. Diagnosis of crime rate against women using k-fold cross validation through machine learning. 2020 fourth international conference on computing methodologies and communication (ICCMC), (2020). 1034–1038.

[ref66] Tareq W. Z. T. Davud M. Classification and clustering. Decision-Making Models. Elsevier. (2024).

[ref67] Ugboke P. Ebimaro J. Olanrewaju P. Orukpe P. (2024). The impact of digitization towards technological revolution in the power industry. J. Energy Technol. Environ. 6, 193–200. doi: 10.5281/zenodo.11420895

[ref68] Ukkelberg M. (2018). Utilisation of machine learning in power transformer asset Management: NTNU.

[ref69] Uwamahoro N. Eftekharnejad S. A comparative study of data-driven power grid cascading failure prediction methods. 2023 North American Power Symposium (NAPS), (2023). 1–6.

[ref70] Vaish R. Dwivedi U. Tewari S. Tripathi S. M. (2021). Machine learning applications in power system fault diagnosis: research advancements and perspectives. Eng. Appl. Artif. Intell. 106:104504. doi: 10.1016/j.engappai.2021.104504

[ref71] Vanwinckelen G. Blockeel H. On estimating model accuracy with repeated cross-validation. BeneLearn 2012: Proceedings of the 21st Belgian-Dutch conference on machine learning (2012) 39–44

[ref72] Wang H. Tan Z. Liang Y. Li F. Zhang Z. Ju L. (2024). A novel multi-layer stacking ensemble wind power prediction model under Tensorflow deep learning framework considering feature enhancement and data hierarchy processing. Energy 286:129409. doi: 10.1016/j.energy.2023.129409

[ref73] Wang Y. Sun Y. Dan Y. Li Y. Cao J. Han X. (2023). Online load-loss risk assessment based on stacking ensemble learning for power systems. Front. Energy Res. 11:1281368. doi: 10.3389/fenrg.2023.1281368

[ref74] Wayahdi M. R. Syahputra D. Ginting S. H. N. (2020). Evaluation of the K-nearest neighbor model with K-fold cross validation on image classification. Info 9, 1–6. doi: 10.25008/ijadis.v2i1.1204

[ref75] Wenyu Z. Hongyong L. Xiaochuan X. Ming L. Weixi R. Buyun M.. (2021). Construction of digital operation and maintenance system for new energy power generation enterprises. E3S Web Conf. 236. doi: 10.1051/e3sconf/202123602017

[ref76] Wu C. Hargreaves C. A. (2021). Topological machine learning for mixed numeric and categorical data. Int. J. Artificial Intell. Tools 30:2150025. doi: 10.1142/S0218213021500251

[ref77] Xu H. Chai L. Luo Z. Li S. (2022). Stock movement prediction via gated recurrent unit network based on reinforcement learning with incorporated attention mechanisms. Neurocomputing 467, 214–228. doi: 10.1016/j.neucom.2021.09.072

[ref78] Yeardley A. S. Ejeh J. O. Allen L. Brown S. F. Cordiner J. (2022). Predictive maintenance in the digital era. Computer Aided Chemical Engineering: Elsevier.

[ref79] Zhang W. Yin T. Zhao Y. Han B. Liu H. (2022). Reinforcement learning for stock prediction and high-frequency trading with T+ 1 rules. IEEE Access 11, 14115–14127. doi: 10.1109/ACCESS.2022.3197165

